# ^18^ F-click labeling and preclinical evaluation of a new ^18^ F-folate for PET imaging

**DOI:** 10.1186/2191-219X-3-68

**Published:** 2013-09-16

**Authors:** Hanno Schieferstein, Thomas Betzel, Cindy R Fischer, Tobias L Ross

**Affiliations:** 1 Institute of Nuclear Chemistry, Johannes Gutenberg University Mainz, Mainz 55128, Germany; 2 Center for Radiopharmaceutical Sciences of ETH, PSI and USZ, Institute of Pharmaceutical Sciences, ETH Zürich, Zurich 8093, Switzerland; 3 Radiochemistry/Radiopharmacy, Department of Nuclear Medicine, Hannover Medical School, Hannover 30623, Germany

**Keywords:** PET, Fluorine-18, Folic acid, Folate receptor, Click chemistry

## Abstract

**Background:**

The folate receptor (FR) is a well-established target for tumor imaging and therapy. To date, only a few ^18^ F-folate conjugates via ^18^ F-prosthetic group labeling for positron emission tomography (PET) imaging have been developed. To some extent, they all lack the optimal balance between efficient radiochemistry and favorable *in vivo* characteristics.

**Methods:**

A new *clickable* olate precursor was synthesized by regioselective coupling of folic acid to 11-azido-3,6,9-trioxaundecan-1-amine at the γ-position of the glutamic acid residue. The non-radioactive reference compound was synthesized via copper-catalyzed azide-alkyne cycloaddition of 3-(2-(2-(2-fluoroethoxy)ethoxy)ethoxy)prop-1-yne and γ-(11-azido-3,6,9-trioxaundecanyl)folic acid amide. The radiosynthesis was accomplished in two steps: at first a ^18^ F-fluorination of 2-(2-(2-(prop-2-yn-1-yloxy)ethoxy)ethoxy)ethyl-4-methylbenzenesulfonate, followed by a ^18^ F-click reaction with the γ-azido folate. The *in vitro*, *ex vivo*, and *in vivo* behaviors of the new ^18^ F-folate were investigated using FR-positive human KB cells in displacement assays and microPET studies using KB tumor-bearing mice.

**Results:**

The new ^18^ F-folate with oligoethylene spacers showed reduced lipophilicity in respect to the previously developed ^18^ F-click folate with alkyl spacers and excellent affinity (*K*_i_ = 1.6 nM) to the FR. Combining the highly efficient ^18^ F-click chemistry and a polar oligoethylene-based ^18^ F-prosthetic group facilitated these results. The overall radiochemical yield of the isolated and formulated product averages 8.7%. *In vivo* PET imaging in KB tumor-bearing mice showed a tumor uptake of 3.4% ID/g tissue, which could be reduced by FR blockade with native folic acid. Although the new ^18^ F-oligoethyleneglycole (OEG)-folate showed reduced hepatobiliary excretion over time, a distinct unspecific abdominal background was still observed.

**Conclusions:**

A new ^18^ F-folate was developed, being available in very high radiochemical yields via a fast and convenient two-step radiosynthesis. The new ^18^ F-OEG-folate showed good *in vivo* behavior and lines up with several recently evaluated ^18^ F-labeled folates.

## Background

Since the folate receptor (FR) is a well-established target in tumor imaging and tumor therapy, many radiofolates and chemotherapeutics based on the natural ligand folic acid have been developed and investigated [1]. Folic acid is a vitamin essential for *de novo* DNA synthesis in eukaryotic cells where it is converted into the co-enzyme 5,6,7,8-tetrahydrofolate and acts as a carrier of C1 building blocks [2]. The FR is a glycosyl phosphatidylinositol-anchored protein which has a high affinity for folic acid (*K*_d_ ~ 1 nM) and is (over)expressed in many types of human tumors, e.g., ovarian cancer or endometrial cancer [3]–[5]. The expression of the FR in healthy tissues, directly accessible from the bloodstream, is limited to the proximal tubules of the kidneys, where it is involved in the recycling of folic acid from renal excretion [6,7]. Hence, specific accumulation of an intravenously administered radiofolate is mostly associated with a pathophysiological cause. Therefore, many folate conjugates, featuring different radionuclides for various applications, have been developed and evaluated in the past two decades [8]. The introduction of FR targeting to tumor diagnostics in the field of nuclear imaging goes back to 1981 using ^125^I-labeled *pteroylglutamic acid* (equals folic acid) [9], which was not particularly promising. In spite of that, a number of radiofolates have been reported, many of which feature radionuclides useful in single photon emission computed tomography. Examples are ^111^In-diethylenetriamine pentaacetic acid-folates, ^99m^Tc-folates, and ^67^Ga-folates, which showed promising results in preclinical *in vivo* tumor targeting [10]–[13]. In 2006, Bettio and co-workers synthesized a ^18^ F-labeled folate for application in positron emission tomography (PET), formed by amide coupling of the prosthetic group 4-[^18^ F]fluorobenzylamine and native folic acid. The coupling afforded a mixture of α- and γ-regioisomers, which was not separated before *in vivo* animal PET studies [14]. Good visualization of FR-positive tumors was achieved; however, one major drawback was the time-consuming multistep radiosynthesis, the regioisomeric mixture, and low radiochemical yields. To overcome the complicated radiosynthesis and provide a regioselective product, another radiofolate was developed using the copper-catalyzed azide-alkyne cycloaddition (CuAAC, click reaction). The structural isomerism was circumvented by a regioselective derivatization at the carboxylic acid in the γ-position of the folate precursor [15]. The radio-CuAAC clearly simplified the radiosynthesis and gave the first ^18^ F-click-labeled folate in high radiochemical yields within ≤90 min. However, *in vivo* animal PET imaging revealed an unfavorable biological distribution profile with a poor signal-to-noise ratio and a very high abdominal background, which were assumed to be related to the loss of hydrophilicity of the tracer. To retain the polarity of the radiofolate, which is obviously necessary for favorable *in vivo* characteristics, a radiofolate was developed by coupling a folate carbohydrazide with 2-[^18^ F]fluoro-2-deoxy-d-glucose ([^18^ F]FDG) via oxime formation with the open-chain form of glucose [16]. Another approach of Fischer and co-workers also used the efficiency of CuAAC for ^18^ F-radiolabeling in combination with the inevitable polarity of [^18^ F]FDG to enhance pharmacodynamics [17]. In this case, a ^18^ F-labeled azido-FDG derivative was used as a prosthetic group and coupled via CuAAC to an alkyne-carrying γ-folate. This derivative gave promising results, with significantly enhanced tumor-to-background ratios due to a high tumor uptake and reduced background. An alternative to folate radioconjugates is the derivatives synthesized by direct ^18^ F-fluorination approaches, which led to the development of 2′-[^18^ F]fluorofolic acid, synthesized via a nucleophilic aromatic ^18^ F-flourination at the 2′-position of folic acid [18]. Preclinical evaluation showed excellent *in vivo* behavior with a clear-cut visualization of FR-positive KB tumors and healthy tissues (kidneys). However, the direct ^18^ F-fluorination of folic acid requires protecting group chemistry with cleavage under harsh conditions, resulting in degradation and poor radiochemical yields. Very recently, an optimized version of 2′-[^18^ F]fluorofolic acid was reported [19]. The intended aromatic ring was exchanged by a pyridine to further reduce the electron density in the 2′-position for the nucleophilic ^18^ F-fluorination. As a result, the radiochemical yield was significantly improved and, besides an increased liver uptake, the pharmacokinetic characteristics were excellent for *in vivo* PET imaging. In respect to the ^18^ F-folates via prosthetic group conjugates, a new ^18^ F-polyethylene glycol (PEG)-folate was developed very recently and published while this manuscript was in preparation. The new ^18^ F-PEG-folate was not primarily intended for tumor imaging, but for targeting the FR-β on activated macrophages in a rat model of arthritis [20]. The major objective was to reduce background signal in the periarticular tissue by following a similar strategy as for the here presented work to improve pharmacokinetics by introducing oligoethylene glycol spacers [21].

The aim of this study was to investigate the influence of oligoethylene glycol spacers on the polarity of a conjugated ^18^ F-labeled radiofolate, when introduced at the γ-position of the glutamate residue, since many drugs showed improved pharmacokinetics due to PEGylation [21]. In this paper, the synthesis, radiolabeling, and preclinical evaluation of a new ^18^ F-labeled γ-radiofolate are reported. The radiofolate features oligoethylene glycol spacers for enhanced polarity and is radiolabeled via the highly efficient radio-CuAAC reaction.

## Methods

### General

Reagents and solvents were purchased from Sigma-Aldrich Co. (St. Luois, MO, USA), Acros (Geel, Belgium), or Merck AG (Darmstadt, Germany) and used without further purification, unless otherwise stated. The building block *N*^2^-*N*,*N*-dimethylaminomethylene-10-formylpteoric acid was generously provided by Merck & Cie AG (Schaffhausen, Switzerland). ^3^H-folic acid was purchased from Moravek Biochemicals Inc. (Brea, CA, USA). Reactions were monitored by thin layer chromatography (performed on Merck silica gel 60 F254, not modified, pre-coated silica gel on aluminum-supported plates) or high-performance liquid chromatography (HPLC).

Radiosyntheses were performed either in a manipulator-equipped hot cell by conventional heating (starting activities >5 GBq [^18^ F]fluoride) or manually in a lead-shielded fume hood (starting activities ≤5 GBq [^18^ F]fluoride) using a focused laboratory microwave (CEM Discover, Matthews, NC, USA) in the following mode: 1-min pre-run, 10-min reaction time, and a maximum power of 300 W.

Information about compound characterizations and analytical or preparative HPLC as well as radio-HPLC conditions can be found in Additional file 1.

Small animal PET imaging was performed on a *GE eXplore Vista PET/CT* scanner (GE Healthcare, Little Chalfont, UK).

#### ***Synthesis of the 2-(2-(2-(prop-2-yn-1-yloxy)ethoxy)ethoxy)ethyl 4-methylbenzenesulfonate (10)***

The 2-(2-(2-(prop-2-yn-1-yloxy)ethoxy)ethoxy)ethyl 4-methylbenzenesulfonate was prepared using a modified method of that described by Li and co-workers [22]. Briefly, 2-[2-(2-hydroxyethoxy)ethoxy]ethanol (**7**) (5 g, 33 mmol) was added to a suspension of sodium hydride (1.3 g, 33 mmol) in dimethylformamide (15 mL) cooled to 0°C, and propargyl bromide (3.5 mL, 33 mmol) was added. The reaction mixture was then allowed to warm to room temperature and stirred. After 24 h, the solvent was removed and the crude reaction mixture purified by silica gel column chromatography (ethyl acetate/*n*-hexane, 1:2) to give **8** as a pale yellow oil in 42% yield (2,6 g, 14 mmol).

Compound **8** (1 g, 5 mmol) and *p*-toluenesulfonyl chloride (1.9 g, 10 mmol) were dissolved in anhydrous dichloromethane (10 mL) and cooled to 0°C. 1,4-Diazabicyclo[2.2.2]octene (560 mg, 5 mmol), dissolved in 5 mL dichloromethane, was added dropwise to the solution. The reaction mixture was stirred for 2 h at room temperature and gave, after purification by column chromatography, **10** as a colorless oil (54%, 940 mg, 2.7 mmol).

#### ***Synthesis of 3-(2-(2-(2-fluoroethoxy)ethoxy)ethoxy)prop-1-yne (9)***

Compound **8** (200 mg, 1 mmol) was dissolved in anhydrous dichloromethane (5 mL) and cooled to 0°C. To this solution, *N*,*N*-diaminosulfur trifluoride (132 μL, 1 mmol) was added, and the reaction mixture maintained at 0°C for 1 h, before allowing it to warm to room temperature and stirring for additional 5 h. After removal of the solvent *in vacuo*, the crude reaction mixture was purified via column chromatography to give **9** as a pale yellow oil (40%, 80 mg, 0.4 mmol).

#### ***Synthesis of γ-(11-azido-3,6,9-trioxaundecanyl)folic acid amide (5)***

*N*-(*tert*-butoxycarbonyl)glutamic acid α-methyl ester (**1**) (200 mg, 0.7 mmol) was reacted with 11-azido-3,6,9-trioxaundecan-1-amine (152 mg, 0.7 mmol) using 298 mg (0.7 mmol) 1-cyano-2-ethoxy-2-oxoethylidenaminooxy)dimethylamino-morpholino-carbenium hexafluorophosphate (COMU) as coupling agent and 2 eq. of 2,2,6,6-tetramethylpiperidine (TMP) as base. The reaction was performed in acetonitrile and stirred at room temperature for 16 h. The crude residue was re-dissolved in dichloromethane (20 mL) and washed successively with aqueous hydrochloric acid (0.1 M, 2 × 10 mL) and aqueous sodium hydrogen carbonate (0.1 M, 3 × 10 mL), dried over sodium sulfate, and filtered, and the solvent was removed under reduced pressure. Purification by silica gel column chromatography (ethyl acetate/hexane, 10:1) afforded a colorless oil (86%, 274 mg, 0.6 mmol). Compound **2** was deprotected using dichloromethane/trifluoroacetic acid (1:1, 10 mL) at room temperature for 12 h. The mixture was co-evaporated with toluene (3 × 5 mL) and used in the next step without further purification. After deprotection, **3** was coupled to the activated ester of protected pteroic acid (230 mg, 0.6 mmol) prepared by adding COMU (256 mg, 0.6 mmol) and TMP (2 eq.) in anhydrous dimethylformamide (5 mL) to give the activated ester complex. The deprotected **3** was added dropwise to the solution, and the mixture was stirred at 40°C for 12 h, after which the solvent was removed *in vacuo*. The crude reaction mixture was re-dissolved in dichloromethane and extracted analogously to the coupling reaction of **1** and 11-azido-3,6,9-trioxaundecan-1-amine. The mixture was purified first by aluminum oxide column chromatography (dichloromethane/methanol, 15:1) followed by a second *flash* chromatography on silica gel (ethyl acetate/methanol, 5:1), giving 103 mg (0.14 mmol) of **4** as a yellow powder.

**Scheme 1:**
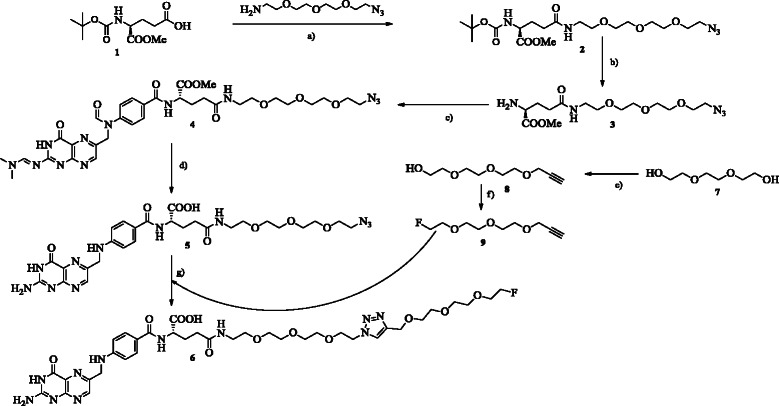
**Synthesis of the azido-folate (5) and the reference compound (6). (a)** COMU, TMP, MeCN, rt. **(b)** DCM/TFA (1:1), rt. **(c)** COMU, TMP, DMF, rt. **(d)** 1 M NaOH (aq.), rt. **(e)** NaH, DMF, 0°C to rt. **(f)** DCM, DAST, 0°C to rt. **(g)** Cu(I)I, sodium ascorbate, MeCN, 0.05 M phosphate buffer, DIPEA/2,6-lutidine (1:1), microwave.

For deprotection of **4**, 30 mg (0.04 mmol) was dissolved in a 1-M sodium hydroxide solution (1 mL) and stirred at room temperature for 16 h. After 16 h, the pH was adjusted to 2 using a 2-M hydrochloric acid solution resulting in the precipitation of the product. After centrifugation, the supernatant was removed and the product was washed twice with water, leading to 19 mg (0.03 mmol) of the final azido-folate **5** (Scheme 1).

Synthesis of the 16-(4-(((2-amino-4-oxo-3,4-dihyxdropteridin-6-yl)methyl)amino)benzamido)-1-(4-(2-(2-(2-fluoroethoxy)ethoxy)ethoxy)-1H-1,2,3-triazol-1-yl)-13-oxo-3,6,9-trioxa-12-azaheptadecan-17-oic acid (6).

Compound **5** (5 mg, 0.02 mmol), copper(I) iodide (0.5 eq.), and a mixture of diisopropylethyl amine (DIPEA)/2,6-lutidine (1 eq.) were dissolved in 2 mL acetonitrile. The reaction was allowed to react for 15 min before 15 mg (0.02 mmol) of **9**, dissolved in 2 mL of 0.05 M phosphate buffer, was added in one portion to the reaction mixture. The mixture was reacted at 130°C for 9 min in a sealed vessel using a laboratory microwave at 55 W and purified by semi-preparative HPLC. The combined fractions were lyophilized and re-dissolved in water (1 mL) and the pH was adjusted to 2, leading to the precipitation of the product, which was separated by centrifugation (10,000 rpm, 8 min). The supernatant was removed and the precipitate was lyophilized, yielding 9 mg (0.01 mmol) of **6** as a yellow solid.

Synthesis of 16-(4-(((2-amino-4-oxo-3,4-dihydropteridin-6-yl)methyl)amino)benzamido)-1-(4-(2-(2-(2-[^18^ F]fluoroethoxy)ethoxy)ethoxy)-1H-1,2,3-triazol-1-yl)-13-oxo-3,6,9-trioxa-12-azaheptadecan-17-oic acid ([^18^ F]12).

No-carrier-added (n.c.a.) [^18^ F]fluoride was produced via the ^18^O(p,n)^18^ F nuclear reaction. Isotopically enriched [^18^O]water (97% enrichment) was irradiated by an 18-MeV proton beam and trapped on an anion exchange resin (Sep-Pak Light Waters Accell Plus QMA Cartridge, Waters Corporation, Milford, MA, USA), which was pre-conditioned with a 1-M potassium carbonate solution (10 mL) and rinsed with pure water (20 mL). The n.c.a. [^18^ F]fluoride was eluted with 1 mL of a methanolic tetrabutylammonium hydroxide solution (95.7 mg tetrabutylammonium hydroxide (TBA-OH) × 30 H_2_O in 2 mL methanol) into a 5-mL sealed reaction vial. After azeotropic drying using three portions of acetonitrile, 800 μL of acetonitrile was added to the dry [^18^ F]fluoride-base mixture. The 2-(2-(2-(prop-2-yn-1-yloxy)ethoxy)ethoxy)ethyl 4-methylbenzenesulfonate **10** (6 mg, 17 μmol) was dissolved in 200 μL acetonitrile and subsequently added to the [^18^ F]fluoride solution. The reaction time was 12 min at 110°C, followed by quenching with 5 mL of 50 mM phosphate buffer. After HPLC purification (*t*_R_([^18^ F]**11**), 15 min) the fraction of the ^18^ F-labeled prosthetic group was diluted by addition of 25 mL of water and trapped on a Phenomenex Strata X-C18 cartridge (Torrance, CA, USA). After washing with 5 mL of water, the final prosthetic group **11** was eluted into a 5-mL reaction vial with 800 μL acetonitrile, which was equipped with copper(II) acetate (1.5 mg in 500 μL), sodium ascorbate (9 mg in 500 μL of 0.05 M phosphate buffer), and **5** (2 mg in 500 μL of 0.05 M phosphate buffer). The click reaction was performed at 110°C. After 13 min, the reaction was quenched with 0.05 M phosphate buffer and filled to a final volume of 5 mL. Purification was performed using the semi-preparative radio-HPLC system described before. The product fraction was acidified by addition of 500 μL of a 1-M hydrochloric acid solution and passed through a Phenomenex Strata X-C cartridge. After washing with 5 mL of water, the final radiotracer was eluted with 2.5 mL phosphate-buffered saline containing 10% ethanol.

#### ***Relative lipophilicity (k’ value)***

The relative lipophilicity of **6** was determined as capacity factor *k*’ (*k*’ = (*t*_retention_ - *t*_solvent_) / *t*_solvent_) by reversed-phase HPLC using a methanol-phosphoric acid buffer eluent system at pH 2. Under these conditions, the retention time (*t*_R_) was 4.49 min, which equals a *k*’ value of 1.12. This method has already been described elsewhere [18].

#### ***In vitro binding affinity assays***

Displacement studies using [^3^H]folic acid and the non-radioactive reference compound **6** were carried out according to the previously described procedure [18].

#### ***In vitro metabolite studies in fetal calf serum***

A solution of [^18^ F]**12** (50 μL, approximately 5 MBq) was incubated with 500 μL of fetal calf serum (FCS) at 37°C and the mixture shaken at 900 rpm. The time points were set between 0 and 90 min using 500 μL of FCS and 50 μL of the radiotracer for each time point. Plasma proteins were precipitated by addition of cold acetonitrile (300 μL), followed by centrifugation (10,000 rpm, 10 min). An aliquot of each time point (15, 30, 60, and 90 min, 100 μL) was injected into the analytical radio-HPLC system for analytics.

#### ***In vivo studies***

All animal experiments were approved by the local veterinary department and complied with Swiss and local laws on animal protection. Female CD-1 nude mice (Charles River, Sulzfeld, Germany) were fed with a folate-deficient rodent diet (Harlan Laboratories, Indianapolis, IN, USA). After an acclimatization period of 5 to 7 days, human KB tumor cells (5 × 10^6^ cells in 0.1 mL sterile phosphate-buffered saline (PBS)) were inoculated subcutaneously on both shoulders of each mouse. Twelve days later, the animals were intravenously injected with [^18^ F]**12** (approximately 5 MBq, 100 μL). Blocking studies were performed with excess folic acid dissolved in PBS (100 μg in 100 μL) injected 5 min prior to [^18^ F]**12**. Animals were euthanized at the indicated time points, and selected organs and tissues were collected, weighed, and measured in a γ-counter. The incorporated radioactivity was expressed as percentage injected dose per gram (%ID/g) of tissue. PET/computed tomography (CT) experiments were performed with a dedicated small-animal PET/CT scanner (eXplore Vista PET/CT, Sedecal, Algete, Spain/GE Healthcare). Animals were intravenously injected with [^18^ F]**12** (approximately 13 MBq, 100 μL). For scanning, mice were anesthetized with isoflurane in an air/oxygen mixture. The PET scans were acquired from 60 to 90 min post-injection (p.i.) followed by a CT. After acquisition, PET data were reconstructed in user-defined time frames, and the fused datasets of PET and CT were analyzed with PMOD software (version 3.4).

## Results and discussion

### Organic chemistry

**Scheme 2:**
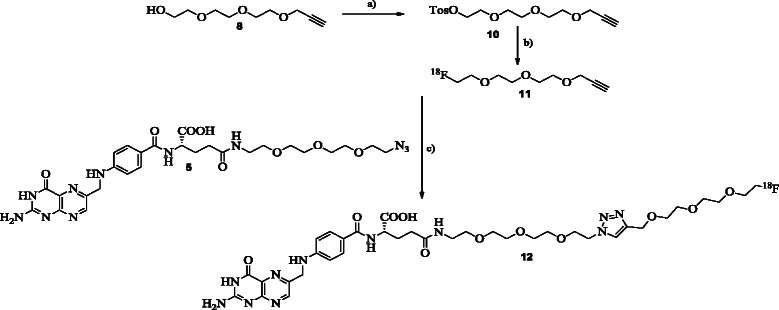
Synthesis of the prosthetic group precursor (10), followed by radiosynthesis of the 18F-labeled prosthetic group [18F]11 and 18F-labeled radiofolate [18F]12. **Synthesis of the prosthetic group precursor (10), followed by radiosynthesis of the **^**18**^ **F-labeled prosthetic group [**^**18**^ **F]11 and **^**18**^ **F-labeled radiofolate [**^**18**^ **F]12. (a)** DCM, TEA, Tos-Cl, rt. **(b)** TBA-OH, acetonitrile, 110°C. **(c)** Cu(II)acetate, sodium ascorbate, 110°C, acetonitrile/water/0.05 M phosphate buffer.

The regioselective buildup synthesis of the γ-azido-folate **5** was straightforward. Starting with the selectively protected glutamic acid **1**, which had its γ-position free for derivatization, it gave the desired glutamic acid derivative **2** after coupling of 11-azido-3,6,9-trioxaundecan-1-amine in good yields of 86% (Scheme 1). The incorporation of the oligoethylene glycol spacer carrying a terminal azido moiety enabled conjugation via the CuAAC reaction, which had previously been applied as an efficient and high-yielding radiolabeling protocol [15]. After Boc removal at the glutamate **2**, it was coupled to protected pteroic acid. As reported by Subiros-Funosas and co-workers [23], a side reaction occurs using COMU as coupling agent. The protected azido-folate **4** was purified in two steps: first by aluminum oxide column chromatography, followed by a second column based on silica gel. As a consequence of two purification steps in particular, the pure product was obtained only in moderate to low yields of 20%. Deprotection of azido-folate (prot.) **4** by addition of a 1-M sodium hydroxide solution, followed by precipitation at pH 2 and washing with pure water, afforded the final azido-folate **5** in 50% yield. The azido-folate **5** was employed as a precursor for the synthesis of the reference compound and the radiotracer. In this respect, the counterpart of the CuAAC reaction, 3-(2-(2-(2-fluoroethoxy)ethoxy)ethoxy)prop-1-yne **9**, was synthesized according to the literature [22]. Triethylene glycol was reacted with propargyl bromide to introduce the alkyne functionality into the prosthetic group for the CuAAC. The free hydroxyl group was either fluorinated via diethylaminosulfur trifluoride (DAST) for the reference compound or tosylated to act as a radiolabeling precursor for the nucleophilic aliphatic ^18^ F-fluorination (Scheme 2). The synthesis of the reference compound was screened in terms of the catalyzing copper species, base, solvent system, and type of heating (Table 1). Optimized conditions comprised Cu(I)I; DIPEA/2,6-lutidine; a mixture of water, buffer, and acetonitrile; and microwave-supported heating. This combination clearly reduced unfavorable side reactions and product degradation during reference compound synthesis.

**Table 1:** Results of the CuAAC reaction for different catalysts and heating conditions

	**Cu(I)I (%)**	**Cu(I)I + sodium**	**Cu(II)acetate + sodium**	**Cu(II)sulfate + sodium**
		**ascorbate (%)**	**ascorbate (%)**	**ascorbate (%)**
Microwave^a^	60 ± 3	22	45	n.d.
Oil bath^b^	20	n.d.	30	15

For all reactions, the used copper concentration was kept constant, whereby that for reactions using copper(II) ascorbate was used in tenfold excess. Yields were determined by HPLC, and for the superior conditions, *n* = 3. ^a^Performed at 110°C for 10 min and a mode with 1-min pre-run and a maximum power of 300 W; ^b^performed at 65°C. n.d., no data.

### Relative lipophilicity (*k*’ value)

The capacity factor *k*’ (*k*’ = (*t*_retention_ - *t*_solvent_) / *t*_solvent_) was determined using reversed-phase HPLC and enables different (radio)folates to be compared based on their polarity. The values determined can be used to give hint to the *in vivo* behavior in terms of the degree of hepatobiliary excretion by comparison with literature examples. The ^18^ F-click folate [15] and the 2-[^18^ F]fluorofolic acid [18] have *k*’ values of 2.28 and 0.53, respectively, of which the latter shows an excellent *in vivo* profile while the former is unfavorable in terms of its abdominal background. The determined *k*’ value of 1.12 for compound **12** was between these two values of the previously synthesized radiofolates, suggesting that it might be a promising candidate for *in vivo* imaging applications.

### Radiochemistry

The established radio-CuAAC approach was used for labeling as it has been proven to produce high radiochemical yields and obviates the need for protecting groups. The prosthetic group [^18^ F]**11** was synthesized by following a modified protocol of Li et al. [22] using tetrabutylammonium hydroxide as base during ^18^ F-fluorination instead of the Kryptofix 2.2.2/potassium carbonate system. In agreement with the findings of Li et al. [22], the radiofluorination showed a strong temperature dependency with a conversion of greater than 75% achieved with conventional heating at 110°C. The crude reaction mixture was purified by semi-preparative HPLC that resulted in 58% radiochemical yield (RCY). After dilution, the prosthetic group [^18^ F]**11** was trapped on a Phenomenex Strata X-C18 cartridge and eluted directly into a vial containing the azido-folate **5**, the copper catalyst, and sodium ascorbate. This final setup was a result of an extensive optimization process, as the conditions used during the non-radioactive reference synthesis could not be successfully translated into radiolabeling. The use of Cu(I)I and a mixture of DIPEA/2,6-lutidine led to significant degradation of the precursor **5** and thus resulted in poor RCYs of 5% to 10%. Therefore, the system described above with no additional base was found optimal. A much higher RCY (≥90%) was achieved through microwave-supported radio-CuAAC; however, such a protocol was not applicable in the manipulator-equipped hot cell used for productions of [^18^ F]**12** for animal studies. For the radiolabeling of the prosthetic group by conventional heating, higher amounts of radioactivity (40 to 100 GBq) were used to compensate for reduced RCY (58%) and prolonged reaction times. Final purification of the radiofolate was achieved using a semi-preparative HPLC system, followed by acidification (pH 1) and subsequent fixation on a Phenomenex Strata X-C cartridge. The loaded cartridge was flushed with water and the product [^18^ F]**12** eluted with PBS buffer containing 10% of ethanol. Sterile filtration of the eluate gave the final product [^18^ F]**12** in a very high radiochemical purity of ≥97% and good overall RCY of 8.7% within a total radiosynthesis time of approximately 2.5 h (end of bombardment (EOB)) for the hot cell-based synthesis. The overall radiosynthesis time could be reduced to approximately 90 min (EOB) using microwave-supported hands-on synthesis in a lead-shielded hood.

#### ***In vitro metabolite studies in fetal calf serum***

The stability of the new radiotracer, [^18^ F]**12**, was evaluated in the presence of FCS. For this purpose, [^18^ F]**12** was added to FCS and incubated at 37°C. At certain time points (15, 30, 60, and 90 min), aliquots were extracted and proteins removed by precipitation. The integrity of the radiotracer was determined by analytical radio-HPLC. No degradation or defluorination of the radiotracer [^18^ F]**12** was observed over 90 min, and therefore, [^18^ F]**12** can be considered stable for the general duration of a microPET (μPET) scan.

#### ***In vitro binding affinity assays***

**Figure 1:**
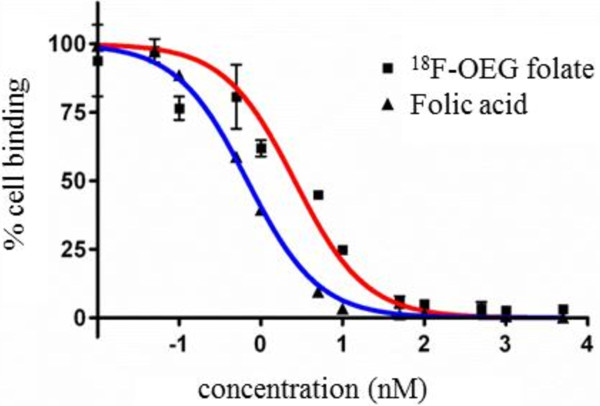
**Displacement assay.** Displacement assay of native folic acid and ^18^ F-OEG-folate **6** using [^3^H]folic acid and human KB cells in cell suspension. Experiments were conducted in triplicate (*n* = 3).

FR-positive human KB cells were kept under folate-deficient conditions to adjust the folate concentration levels to that of the human serum [24]. The half maximal inhibitory concentration (IC_50_) values of native folic acid and the reference compound **6** were determined by displacement studies using [^3^H]folic acid. The displacement curves indicated a slightly higher IC_50_ value for **6** of 3.1 nM (*n* = 3) compared to that of native folic acid (0.9 nM (*n* = 3)) (Figure 1). Using the Cheng-Prusoff equation [25], a *K*_i_ value of 1.6 nM was calculated, assuming a *K*_d_ value for [^3^H]folic acid of 1 nM [4,5,26]. This value is in the very low nanomolar range and close to the *K*_d_ value of native folic acid (approximately 1 nM), indicating a very high affinity of **6** to the folate receptor.

#### ***Ex vivo biodistribution studies***

**Figure 2:**
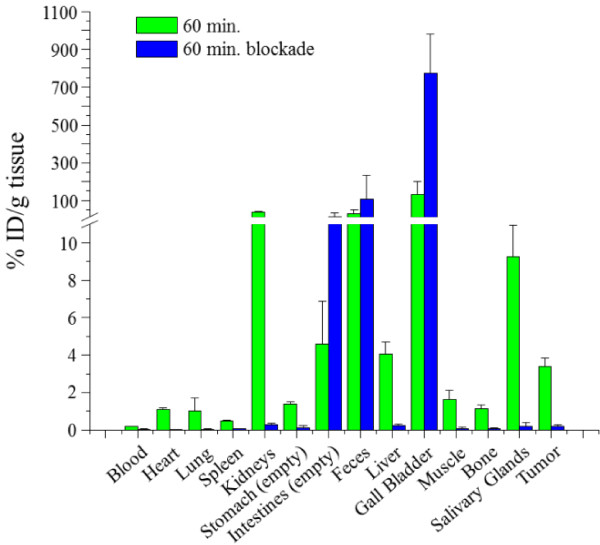
**Results of *ex vivo *biodistribution studies under control and blockade conditions using [**^**18**^ **F]12 in mice.** The human KB tumor-bearing nude mice were euthanized 60 min post-injection. Data are expressed as percentage injected dose per gram tissue (%ID/g). In the blockade group (*n = 3*), 100 μg of native folic acid was injected into each animal 5 min prior to the radiotracer administration, whereas the control group (*n* = 3) received a corresponding volume of PBS.

Biodistribution studies of the new ^18^ F-folate [^18^ F]**12** were performed using human KB tumor-bearing nude mice, determined at 30, 60, and 90 min post-injection. Additionally, a blocking experiment after 60 min using native folic acid was done, whereby 100 μg of native folic acid in PBS buffer (100 μL) was injected 2 min before radiotracer administration. At 30 min post-injection, it was found that the tracer [^18^ F]**12** accumulated in the tumor (3.4%ID/g) and in the kidneys (42%ID/g), remaining constant over 90 min (Table 2). Selective blocking of the folate receptors led to a decrease in specific radiotracer accumulation of 94% in KB tumors and 99% in kidneys (Figure 2). The strong blocking effect reflected the high specificity of the tracer to the FR *in vivo*. Unspecific liver uptake decreased from 5.2%ID/g to 2.1%ID/g over 90 min similar to the background radioactivity in the feces and gall bladder (Table 2), which is believed to be a beneficial effect of the increased polarity of this novel radiofolate. Compared to the biodistribution profile at 45 min post-injection of the former ^18^ F-click folate by Ross and co-workers, the specific uptake of the original ^18^ F-click folate in KB tumors was retained, whereas the specific kidney uptake more than doubled. This indicated a stronger tendency to renal excretion, more likely a result of the increased polarity of the new ^18^ F-folate [^18^ F]**12**. Additionally, the highest unspecific background accumulation in the gall bladder was reduced by half. This background reduction correlated with the more renal excretion and the two times lower *k*’ value as an indicator of lipophilicity of the here presented radiofolate [15]. Similarly, the unspecific accumulation in the liver, feces, and empty intestine was noticeably reduced compared to that of the ^18^ F-click folate. On the other hand, the still increased uptake levels in the gall bladder and feces were evidence of a prominent hepatobiliary elimination pathway of [^18^ F]**12**. However, distinct changes in the molecular structure of the folate-based radioconjugate [^18^ F]**12** induced a higher polarity into the lead molecule ^18^ F-click folate and a significant reduction of the *in vivo* background. The new ^18^ F-folate still showed abdominal background levels, which might impede *in vivo* PET imaging in this region. However, as our results and very recent results of the new and similar ^18^ F-PEG-folate demonstrated [20], such radiofolates have high potential for *in vivo* PET imaging of FR-positive tissues.

**Table 2:** ***Ex vivo *biodistribution studies of **^**18**^ **F-OEG-folate 12 in mice at various time points and **^**18**^ **F-click folate**

	**30 min p.i.**	**60 min p.i.**	**90 min p.i.**	**60 min p.i. blockade**^**a**^	^***18***^ ***F-click folate *45 min p.i.**^**b**^
	**(*n*** **= 3)**	**(*n*** **= 3)**	**(*n*** **= 3)**	**(*n*** **= 3)**	**(*n*** **= 4)**
%ID/g					
Blood	0.20 ± 0.02	0.18 ± 0.01	0.16 ± 0.02	0.04 ± 0.01	0.13 ± 0.01
Heart	1.81 ± 0.61	1.09 ± 0.07	0.79 ± 0.16	0.02 ± 0.01	0.90 ± 0.22
Lung	1.21 ± 0.15	1.02 ± 0.7	0.86 ± 0.17	0.04 ± 0.03	0.85 ± 0.04
Spleen	0.53 ± 0.14	0.49 ± 0.04	0.43 ± 0.08	0.06 ± 0.03	0.33 ± 0.12
*Kidneys*	*41.80 ± 2.58*	*40.77 ± 4.34*	*41.04 ± 7.04*	*0.26 ± 0.08*	*16.53 ± 2.22*
Stomach (empty)	2.18 ± 0.76	1.39 ± 0.13	0.96 ± 0.37	0.12 ± 0.11	2.50 ± 0.6
Intestines (empty)	23.52 ± 7.37	4.56 ± 2.31	1.92 ± 0.52	14.72 ± 19.32	19.59 ± 5.26
Feces	164.2 ± 72.5	29.48 ± 19.04	11.36 ± 4.29	105.5 ± 128.7	56.00 ± 27.64
Liver	5.24 ± 1.57	4.05 ± 0.67	2.30 ± 0.54	0.21 ± 0.09	1.71 ± 0.14
Gall bladder	309.3 ± 187.6	133.2 ± 67.38	55.13 ± n.d.	775.5 ± 206.5	667.4 ± 530.1
Muscle	1.91 ± 0.25	1.64 ± 0.46	1.17 ± 0.03	0.08 ± 0.09	n.d.
Bone	1.47 ± 0.05	1.13 ± 0.21	0.84 ± 0.16	0.06 ± 0.06	0.13 ± 0.01
Salivary glands	8.01 ± 1.16	9.28 ± 1.68	7.03 ± 1.98	0.20 ± 0.18	n.d.
*Tumor*	*3.39 ± 0.54*	*3.39 ± 0.44*	*3.54 ± 0.68*	*0.19 ± 0.07*	*3.13 ± 0.83*
Ratio of tumor to organ or tissue					
Blood	16.81 ± 3.13	18.95 ± 2.68	22.72 ± 6.73		24,08 ± 0.82
Liver	0.69 ± 0.22	0.86 ± 0.19	1.55 ± 0.15		1.18 ± 0.69
*Kidney*	*0.08 ± 0.02*	*0.08 ± 0.01*	*0.09 ± 0.01*		*0.19 ± 1.38*

^a^In the blockade group, each animal received 100 μg/100 μL of folic acid in PBS 5 min before radiotracer injection; ^b^biodistribution data of the ^18^ F-click folate were taken from [15]. n.d., no data. Italicized entries reflect folate receptor-positive tissues/organs.

#### ***In vivo μPET studies***

**Figure 3:**
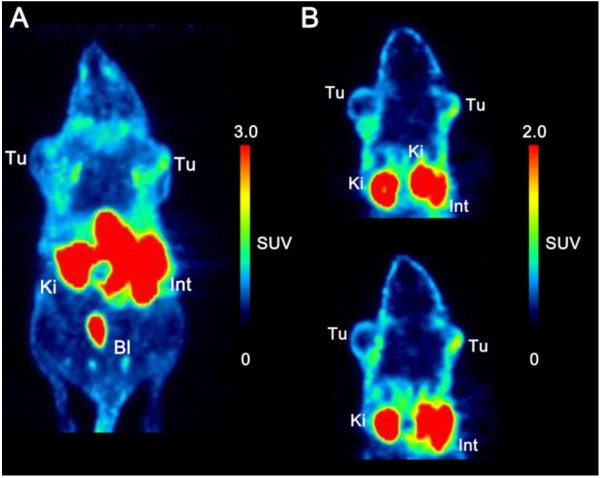
**PET images of a KB tumor-bearing mouse.** Scanned 60 to 90 min after injection of [^18^ F]-OEG-folate **12** (approximately 13 MBq). **(A)** Maximal intensity projection. **(B)** Two representative coronal slices. Tu, tumor; Ki, kidneys; Bl, urinary bladder; Int, intestines/feces; SUV, standardized uptake value.

μPET studies were performed using KB tumor-bearing mice, in which tumors were located in the area above the left and right shoulders. The animals were scanned 60 to 90 min after radiotracer injection to fade out noise signals from perfusion and renal excretion in order to get a higher tumor-to-background contrast. As expected from *ex vivo* biodistribution studies, the kidneys and abdomen (gall bladder, intestines, liver) showed the highest tracer uptake. This caused a poor tumor-to-background contrast in the maximum intensity projection (Figure 3). Tumors are clearly visible in selected coronal slices of μPET scans. In agreement with findings of earlier investigations [14,15], the tracer accumulation was observed only in the outer rim of tumors, indicating that the tumor perfusion is very heterogeneous and the blood supply into the tumor center is believed to be hindered or even blocked. These results are assumed to be due to a high internal tumoral pressure or even a necrotic tumor center of such fast growing xenograft models as already demonstrated in previous studies [27]–[29]. Increased radioactivity accumulation was observed in the abdominal area, which led to an elevated background. This unspecific accumulation is mainly due to the pronounced hepatobiliary excretion. Despite this prominent background, [^18^ F]**12** showed a significantly reduced hepatobiliary excretion compared to the previously synthesized ^18^ F-click folate and most importantly facilitated visualization of FR-positive tumors on the maximum intensity projection. The elevated abdominal background points to a lipophilicity of [^18^ F]**12** not yet being optimal, although the HPLC capacity factor (*k*’ value) of the non-radioactive reference compound **6** indicated that. The uptake in the gall bladder, liver, feces, and intestine could strongly be reduced compared to the original ^18^ F-click folate [15].

## Conclusions

There is a demand for ^18^ F-labeled radiofolates with superior *in vivo* behavior, which can be produced using a facile and robust radiosynthesis transferrable into routine productions for clinical applications. To this end, a novel ^18^ F-radio folate, with high affinity to the folate receptor and increased polarity compared to the original lead compound (^18^ F-click folate), was developed. ^18^ F-labeling of the prosthetic group and the radio-CuAAC reaction were optimized to give excellent RCYs and purities within short reaction times. ^18^ F-click chemistry, by providing a facile and robust labeling procedure, again confirmed its outstanding potential and its particular suitability for ^18^ F-labeling of folate derivatives. *Ex vivo* biodistribution experiments showed a highly specific uptake in FR-positive human KB tumors and kidneys. Compared to the previously developed ^18^ F-click folate, the new radiofolate [^18^ F]**12** showed significantly reduced hepatobiliary excretion while maintaining the tumor uptake. In *in vivo* μPET studies, human KB tumor xenografts were visualized, while a moderate tumor-to-background contrast was found for [^18^ F]**12**. Coronal slices of the PET imaging clearly showed a heterogeneous uptake of [^18^ F]**12** in the outer rim of KB xenografts. In comparison to the previously developed [^18^ F]fluoro-deoxy-glucose folate, the new ^18^ F-OEG-folate showed similar background levels. On the other hand, the threefold higher tumor uptake of the [^18^ F]fluoro-deoxy-glucose folate with significantly increased contrast values led to a much better tumor visualization. However, the very recently reported ^18^ F-PEG-folate with structural similarities to the new ^18^ F-OEG-folate gave promising results in imaging FR-expressing activated macrophages in inflammatory diseases. This study confirms the suitability of such PEGylated ^18^ F-folates for *in vivo* PET imaging of FR-positive tissue and their broad potential. In summary, the newly developed ^18^ F-labeled radiofolate has excellent radiochemical availability and exhibits a high and specific affinity to the folate receptor.

## Competing interests

The authors declare that they have no competing interests.

## Authors’ contributions

HS developed and carried out the organic syntheses and radiochemical syntheses and composed the manuscript. TB performed the radiochemical syntheses (hot cell). CRF performed the microPET analysis. TLR is the project leader and coordinator. All authors read and approved the final manuscript.

## Supplementary Material

Additional file 1 **Supplementary data.** Analytical data of the compounds, radiochemistry, and biological evaluation. Click here for file
