# Supplementary material for: 18 F-click labeling and preclinical evaluation of a new 18 F-folate for PET imaging
Source: EJNMMI Res. 2013 Sep 16;3:68. doi: 10.1186/2191-219X-3-68 (PMC3849131; doi:10.1186/2191-219X-3-68)
Supplement: Additional file 1 — Supplementary data. Analytical data of the compounds, radiochemistry, and biological evaluation. [file 2191-219X-3-68-S1.pdf]

^{18}F-click labeling and preclinical evaluation of a new ^{18}F-folate for PET imaging

H. Schieferstein [a,*], T. Betzel [b], C.R. Fischer [b], T.L. Ross [a,*]

[a] Institute of Nuclear Chemistry, Johannes Gutenberg-University, 55128 Mainz, Germany

[b] Center for Radiopharmaceutical Sciences of ETH, PSI and USZ, Institute of Pharmaceutical Sciences, ETH Zurich, Zurich, Switzerland

Keywords: PET, fluorine-18, folic acid, folate receptor, click chemistry

* Corresponding author. Institute of Nuclear Chemistry, Johannes Gutenberg-University, 55128 Mainz, Germany. Tel.: +49 (0)6131 39-25316; fax +49 (0)6131 39-24510.

Table of contents

ORGANIC CHEMISTRY 4

^{1}H and ^{13}C nuclear magnetic resonance experiments

Low-resolution mass spectra (LR-MS) and high-resolution mass spectra (HR-MS)

High-Pressure Liquid Chromatography

2-(2-(2-(prop-2-yn-1-yloxy)ethoxy)ethoxy)ethanol

Figure 1. ^{1}H NMR; 300MHz, $CDCl_3$

3-(2-(2-(2-fluoroethoxy)ethoxy)ethoxy)prop-1-yne

Figure 2. ^{1}H NMR; 300MHz, $CDCl_3$

2-(2-(2-(prop-2-yn-1-yloxy)ethoxy)ethoxy)ethyl 4-methylbenzenesulfonate

Figure 3. ^{1}H NMR; 300MHz, $CDCl_3$

Figure 4. FD-MS; CH_2Cl_2

N-(tert.-butoxycarbonyl)glutamic acid α-methyl ester

Figure 5. ^{13}C NMR; 300MHz, $CDCl_3$

Figure 6. ^{1}H NMR; 300MHz, $CDCl_3$

N2-N,N-dimethylaminomethylene-10-formyl-α-O-methyl-γ-(11-azido-3,6,9-trioxaundecanyl) folic acid amide11-azido-3,6,9-trioxaundecanyl

Figure 7. 1H NMR; 400MHz, CDCl3

Figure 8. 13C NMR; 300MHz, CDCl3

Figure 9. DEPT-135 NMR; 400MHz, $CDCl_3$

Figure 10. ESI-MS^{+}; MeCN

γ-(11-azido-3,6,9-trioxaundecanyl))folic acid amide

Figure 11. ^{1}H NMR; 400MHz, $CDCl_3$

Figure 12. ^{13}C NMR; 400MHz, $CDCl_3$

Figure 13. DEPT-135 NMR; 400MHz, $CDCl_3$

Figure 14. ESI-MS^{+}; NH_4HCO_3-Buffer (0.05 M)

Figure 15. ESI-MS^{+} (high resolution); NH_4HCO_3-buffer (0.05 M)

16-(4-(((2-amino-4-oxo-3,4-dihydropteridin-6-yl)methyl)amino)benzamido)-1-(4-(2-(2-(2-fluoroethoxy)ethoxy)ethoxy)-1H-1,2,3-triazol-1-yl)-13-oxo-3,6,9-trioxa-12-azaheptadecan-17-oic acid

Figure 16. ^{1}H NMR; 400MHz, DMSO-d_6

Figure 17 ^{13}C NMR; 400MHz, DMSO-d_6

Figure 18. ESI-MS^+; Na/K-phosphate-buffer (0.05 M)

RADIOCHEMISTRY 20

Figure 19. Optimization of the radiolabeling of the prosthetic group

BIOLOGY 23

Figure 20. Plasmastability tests using fetal calve serum

Organic Chemistry

^{1}H and ^{13}C nuclear magnetic resonance experiments

^{1}H and ^{13}C nuclear magnetic resonance spectra were recorded on either a Bruker 300 MHz or 400 MHz spectrometer. Chemical shifts (δ) to solvent are reported in parts per million (ppm) relative to tetramethylsilane and referenced; the following abbreviations are used in the experimental section for the description of ^{1}H-NMR spectra: singlet (s), doublet (d), triplet (t), multiplet (m), broad singlet (br). The chemical shifts of complex multiplets are given as the range of their occurrence.

Low-resolution mass spectra (LR-MS) and high-resolution mass spectra (HR-MS)

Low-resolution mass spectra (LR-MS) and high-resolution mass spectra (HR-MS) were recorded on either a Micromass Quattro micro API LC-ESI or a Finnigan MAT90-Spectrometer.

High-Pressure Liquid Chromatography

For analysis and purification via radio-HPLC, a 50 mM phosphate buffer at pH 7.4 was used (11.29 mmol/L $NaH_2PO_4 \times H_2O$ and 38.71 mmol/L $Na_2HPO_4 \times 2H_2O$). Analytical radio-HPLC was performed on an Agilent 1100 series HPLC system, equipped with a 100 µL loop and a GabiStar radiodetector (Raytest). A reversed-phase column (Gemini C18, 5 µm, 4.6 × 250 mm, Phenomenex®) was used at a flow rate of 1 mL/min with a method as follows: 0-5 min 15% **B** (isocratic), 5-50 min 15-50% **B** (gradient) with **A**=50 mM phosphate buffer and **B**=methanol).

For semipreparative purification, a semi-preparative radio-HPLC was used, equipped with a Smartline Pump 1000, Smartline Manager 5000, Smartline UV-detector 2500 (Knauer), GabiStar radiodetector (Raytest) and a 5 mL loop. A reversed-phase column (Gemini C18, 5 µm, 10 × 250 mm, Phenomenex®) was used. The labeled synthon, [^{18}F]fluoroethoxy)ethoxy)ethoxy)-1H-1,2,3-triazol-1-yl)-13-oxo-3,6,9-trioxa-12-azaheptadecan-17-oic acid ([^{18}F]**11**) was purified, using a flow rate of 3.6 mL/min under isocratic conditions (30% **B**).

For the purification of the radiotracer ^{18}F-OEG folate the following method was used at a flow of 5 mL/min: 0-5 min 100% **A** (isocratic), 5-25 min 0-55% **B** (gradient), 25-35 min 55% **B** (isocratic).

Determination of relative lipophilicity was performed on Dionex ICS-5000 system equipped with a photo diode detector (PDA) using a reversed-phase (Gemini 5*µ* C18 250 × 4.6 mm, Phenomenex®) column and an isocratic solvent system as follows: 80% aqueous solution (pH 2 with *ortho*phosphoric acid) and 20% acetonitrile.

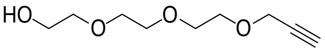

^{1}H NMR (300 MHz, $CDCl_3$) δ 4.16 (d, 2H), 3,71-3.62 (m, 10H), 3.57 (m, 2H), 2.60 (br, 1H), 2.41 (t, 1H) ppm.

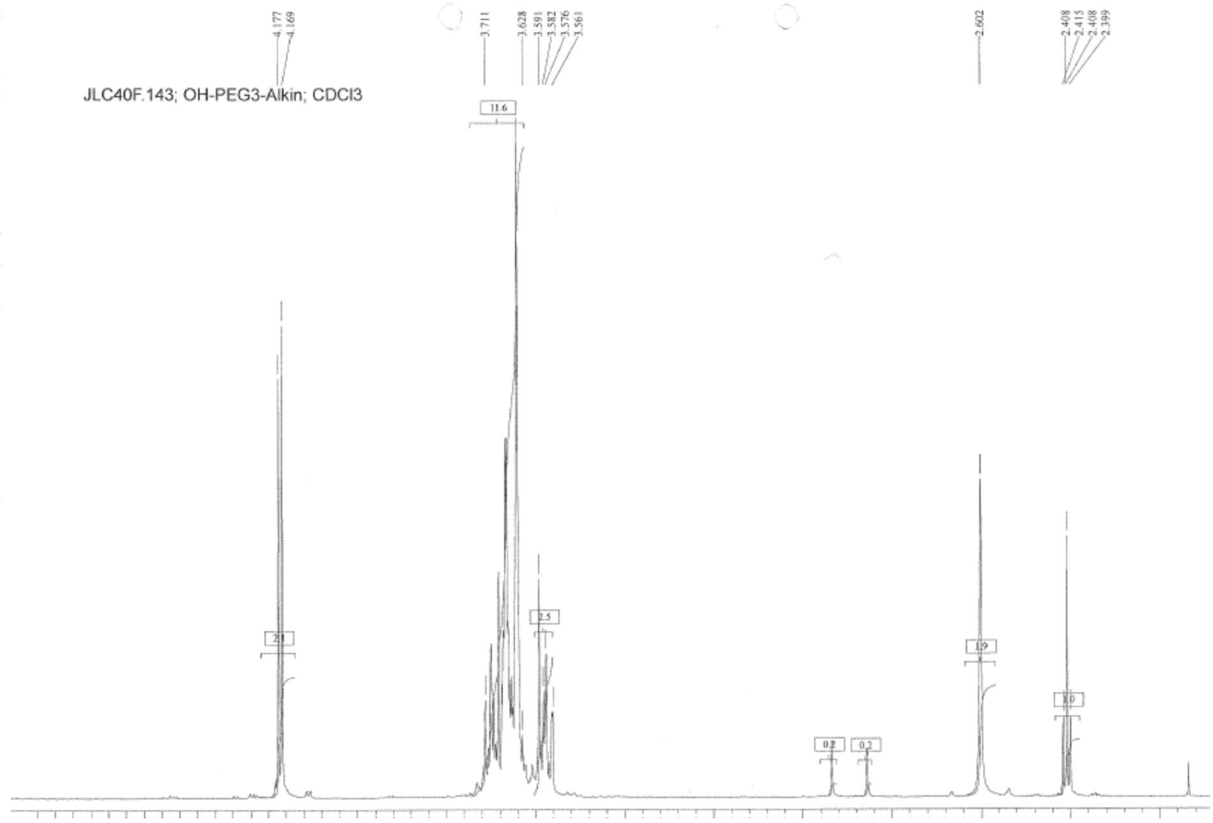

Figure 1. ^{1}H NMR; 300MHz, $CDCl_3$

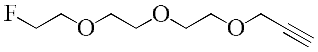

^{1}H NMR (300 MHz, $CDCl_3$) δ 4.61 (t, 1H), 4.44 (t, 1H), 4.17 (d, 2H), 3.67-3.65 (m, 10H), 2.40 (t, 1H) ppm. LR-MS: $[M+H]^+$ = 191.5 (calc. for $C_9H_{15}FO_3$: 191.1).

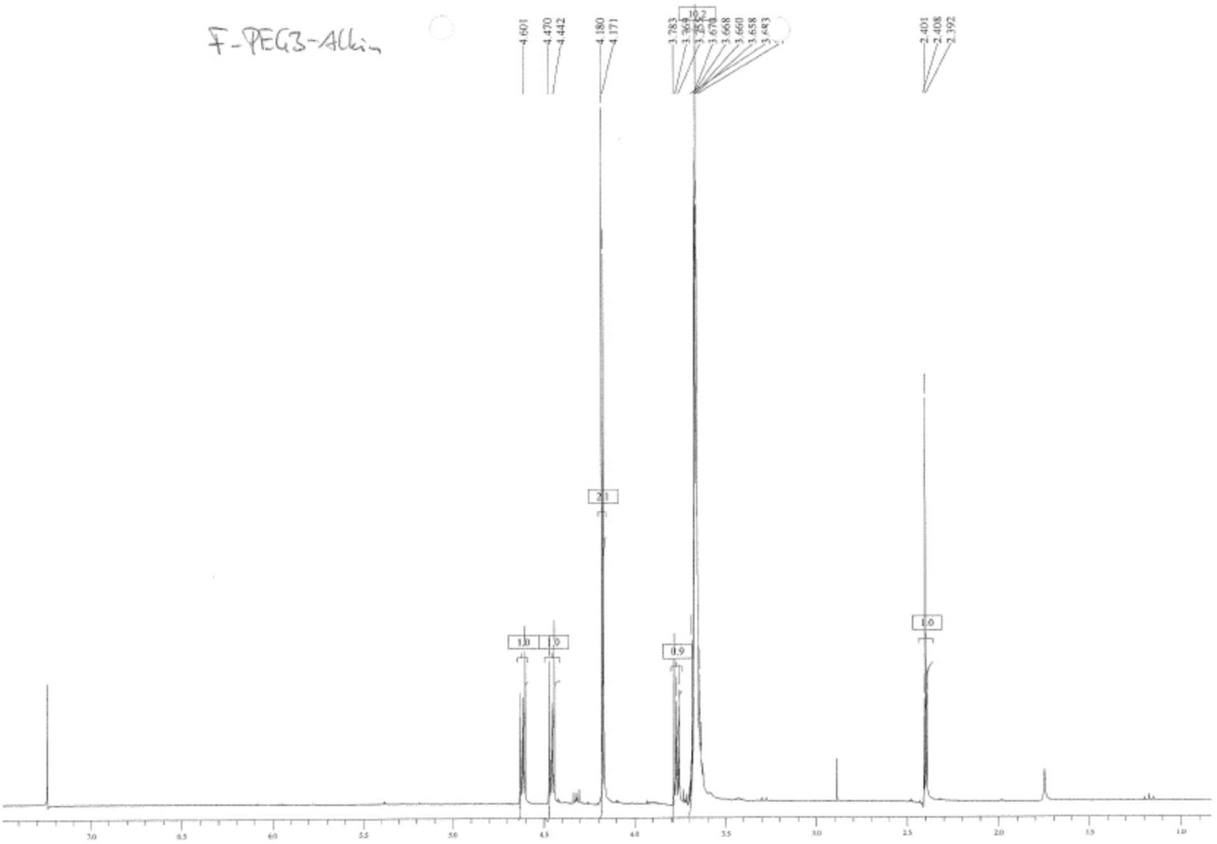

Figure 2. ^{1}H NMR; 300MHz, $CDCl_3$

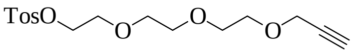

^{1}H NMR (300 MHz, $CDCl_3$) δ 7.75 (d, 2H), 7.30 (d, 2H), 4.15-4.11 (m, 4H), 3.67-3.59 (m, 6H), 2.41 (s, 3H), 2.40 (t, 1H) ppm. ^{13}C NMR (75 MHz, $CDCl_3$) δ 132.96, 129.79, 127.96, 79.59, 74.54, 70.70, 70.53, 70.41, 69.21, 69.04, 68.65, 58.36, 21.61 ppm. LR-MS: $[M+H]^{+}$ = 343.5 (calc. for $C_{16}H_{22}O_6S$: 343.1).

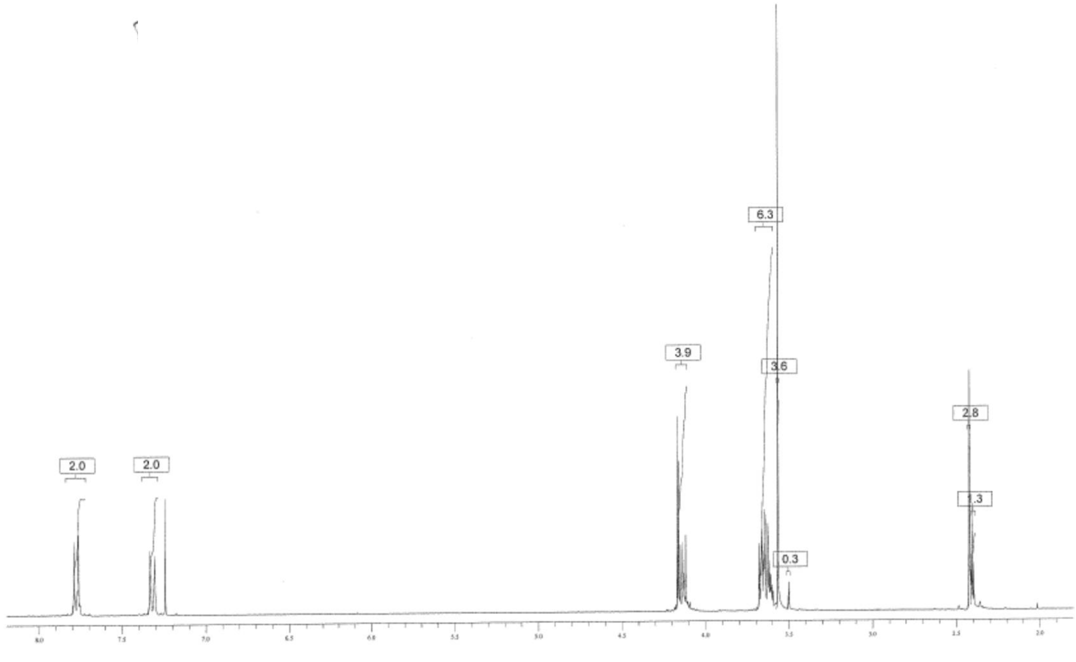

Figure 3. ^{1}H NMR; 300MHz, $CDCl_3$

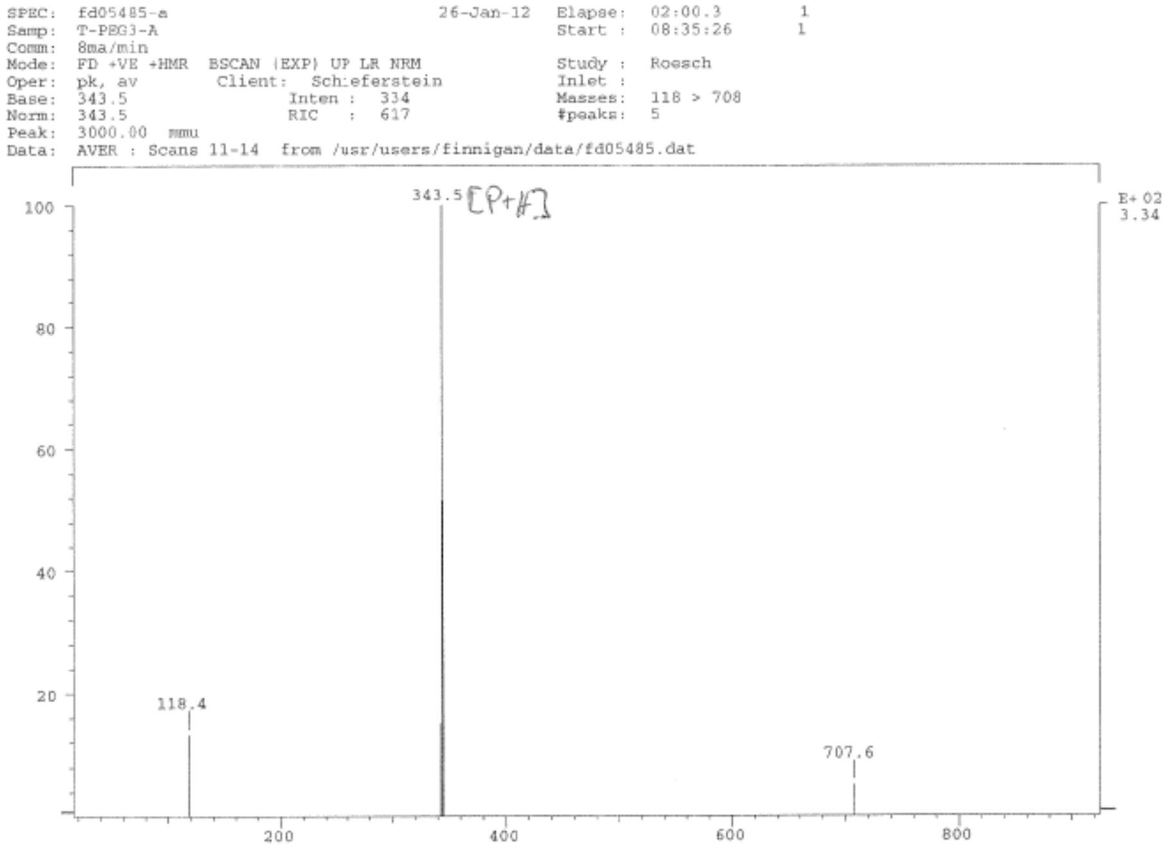

Figure 4. FD-MS; CH_2Cl_2

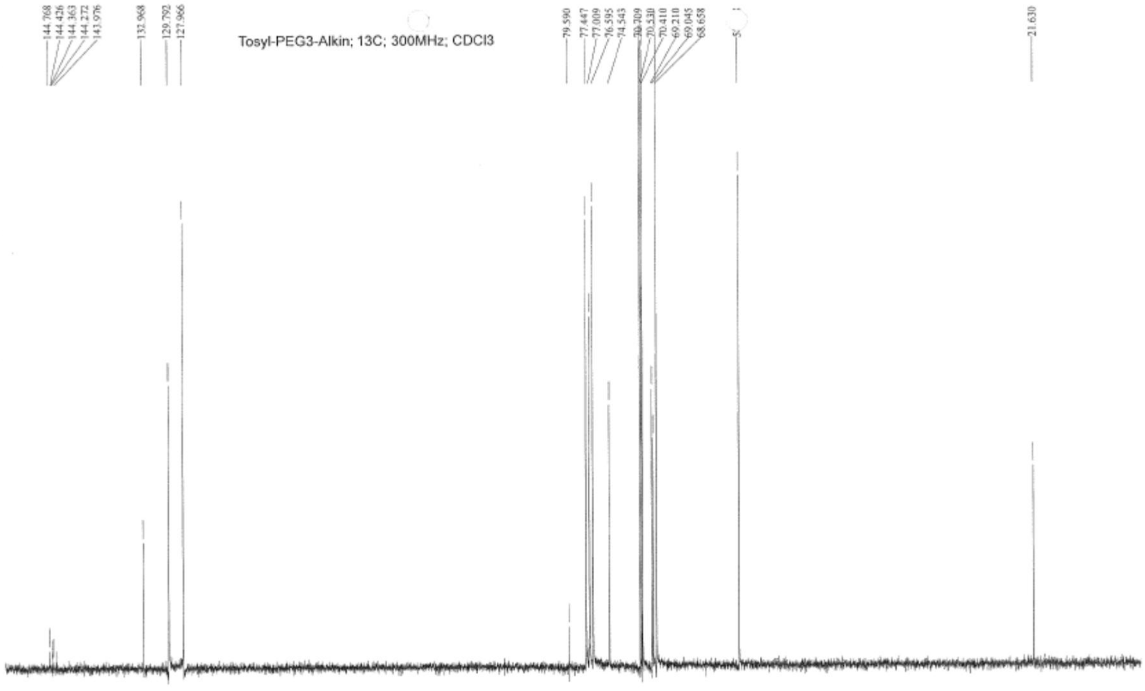

Figure 5. ^{13}C NMR; 300MHz, $CDCl_3$

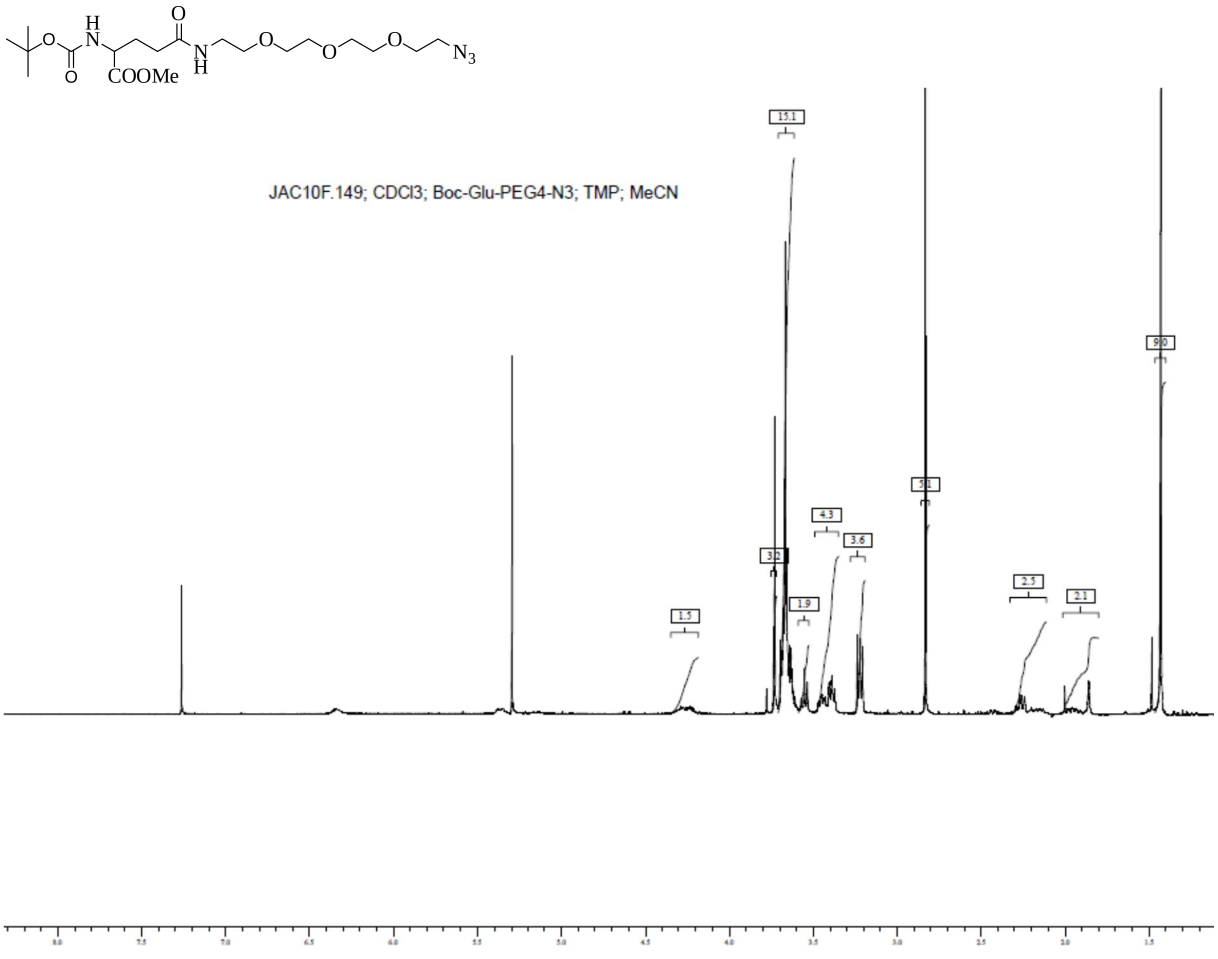

Figure 6. ^{1}H NMR; 300MHz, $CDCl_3$

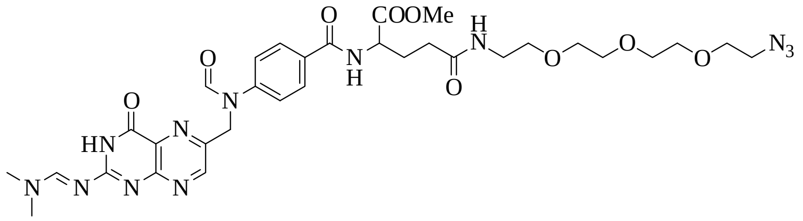

^{1}H NMR (400 MHz, $CDCl_3$) δ 8.91 (s, 1H), 8.70 (d, 2H), 7.87 (d, 2H), 7.30 (d, 2H), 5.29 (s, 2H), 4.61-4.56 (m, 1H), 3.70 (s, 3H), 3.60-3.40 (m, 12H), 3.36 (m, 2H), 3.34 (t, 2H), 3.19 (s, 3H), 3.12 (s, 3H), 2.45-2.32 (m, 2H), 2.20-2.13 (m, 2H) ppm. LR-MS: $[M+H]^+$ = 739.36, $[M+Na]^+$ = 761.33 (calc. for $C_{32}H_{42}N_{12}NaO_9$: 761.31).

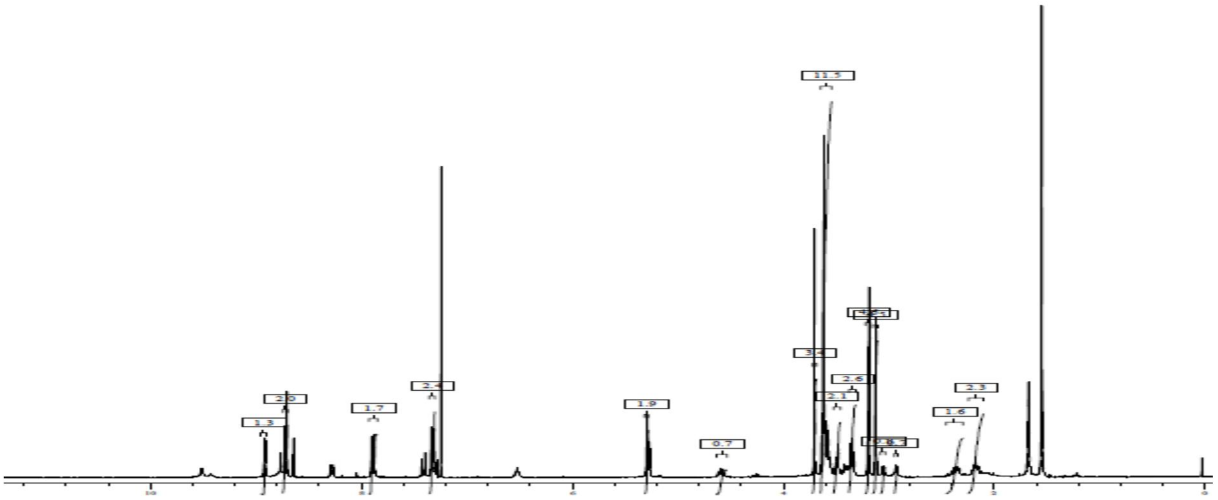

Figure 7. ^{1}H NMR; 400MHz, $CDCl_3$

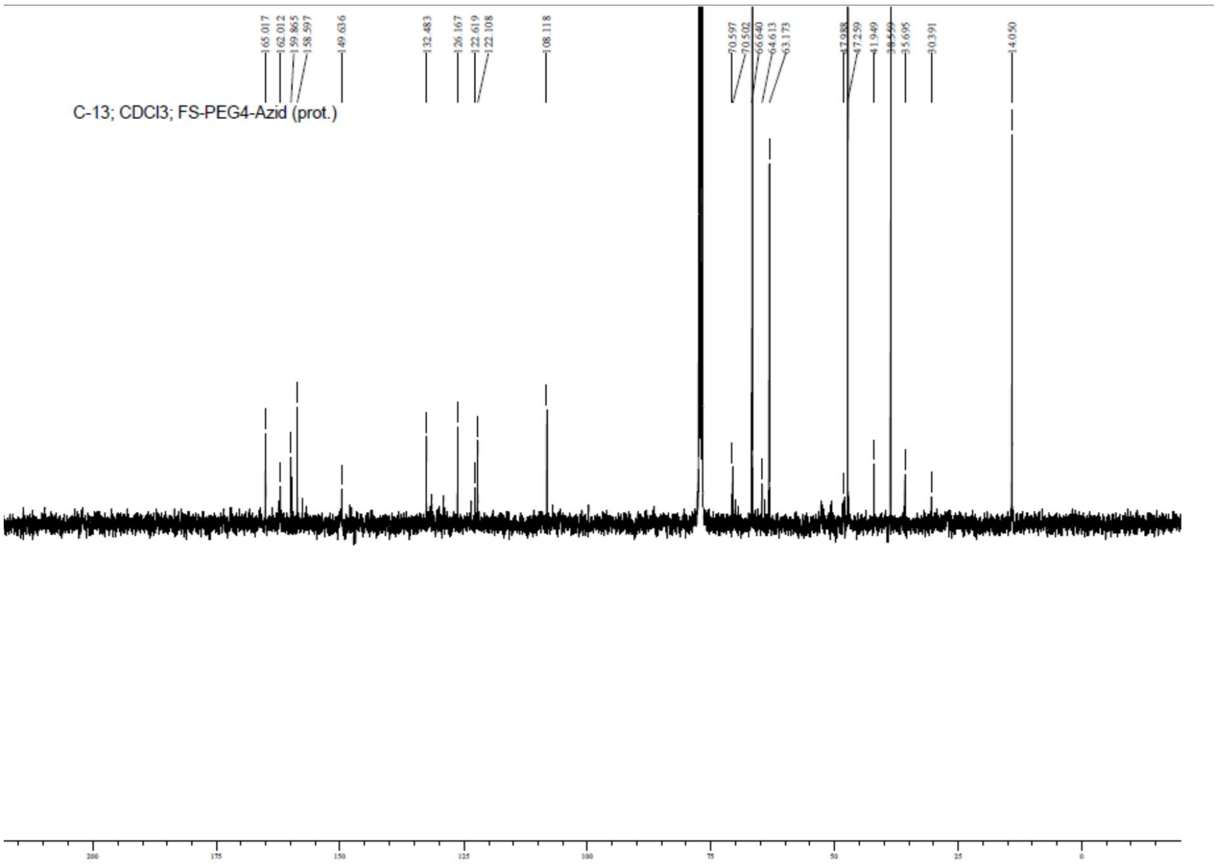

Figure 8. ^{13}C NMR; 300MHz, $CDCl_3$

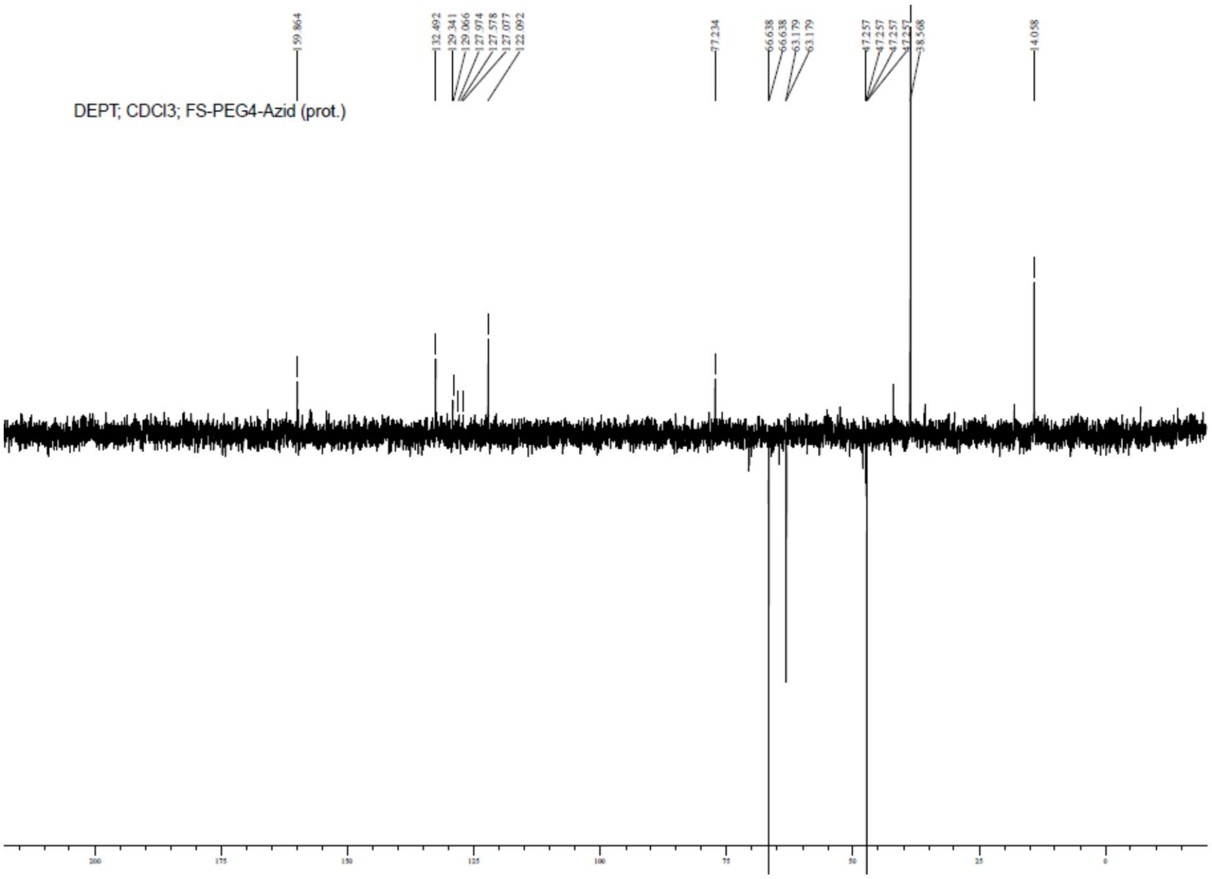

Figure 9. DEPT-135 NMR; 400MHz, $CDCl_3$

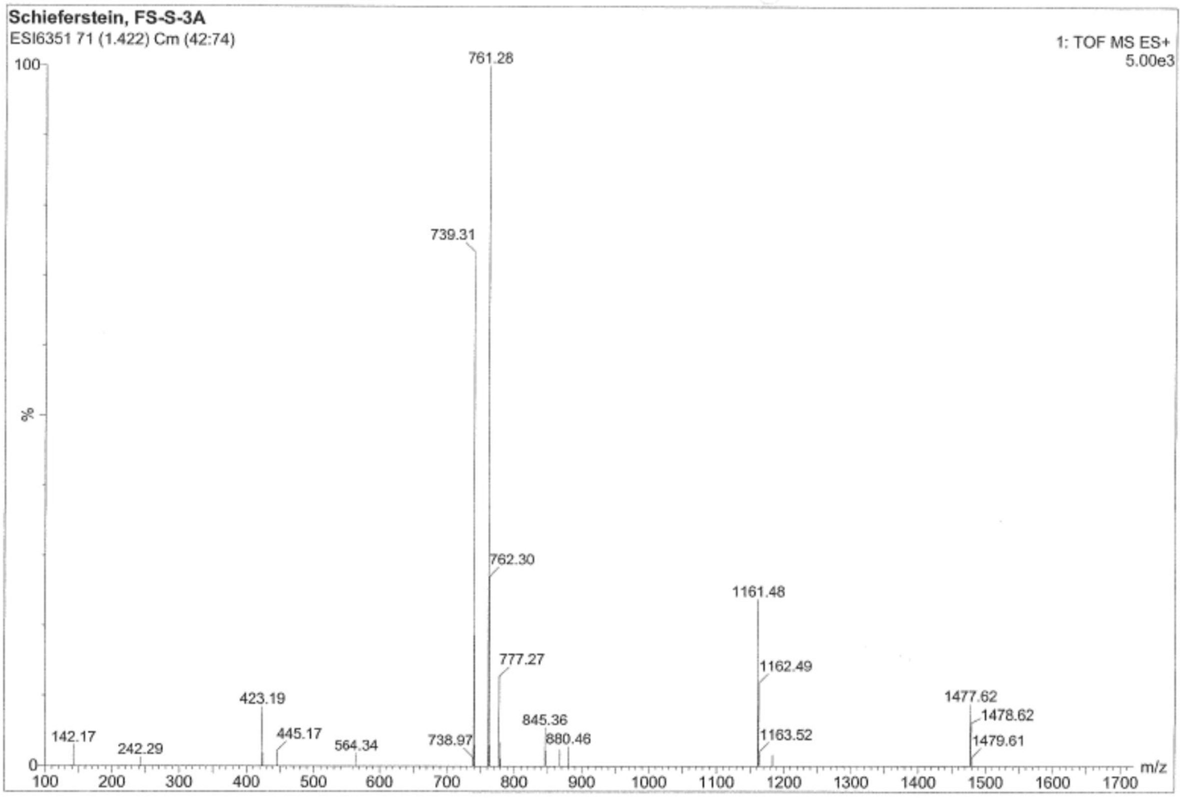

Figure 10. ESI-MS$^+$; MeCN

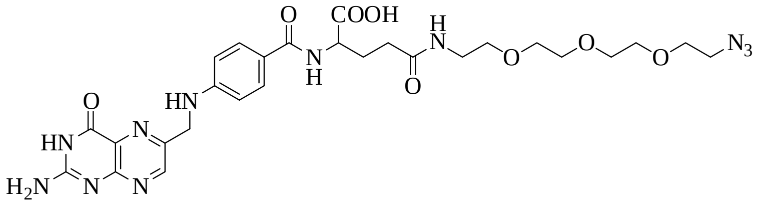

^{1}H NMR (400 MHz, DMSO-d_6) δ 11.52 (br, 1H), 8.63 (s, 1H), 7.62 (d, 2H), 6.61 (d, 2H), 4.46 (s, 2H), 4.26-4.24 (m, 1H), 3.57 (t, 2H), 3.52-3.35 (m, 10H), 3.17 (m, 2H), 2.18 (m, 2H), 2.06-1,99 (m, 1H), 1.93-1.83 (m, 1H) ppm. ^{13}C NMR (100 MHz, DMSO-d_6) δ 174.32, 172.14, 166.70, 154.26, 151.21, 149.13, 129.59, 129.41, 128.41, 121.80, 111.61, 72.79, 70.22, 70.17, 70.12, 70.0, 69.69, 60.65, 52.70, 50.42, 46.36, 32.40, 27.04 ppm. $[M+Na]^+$ = 664.28 (calc. for $C_{27}H_{35}N_{11}NaO_8$: 664.26) HR-MS: $[M+H]^+$ = 642.2746 (calc. for $C_{27}H_{35}N_{11}O_8$: 642.2748).

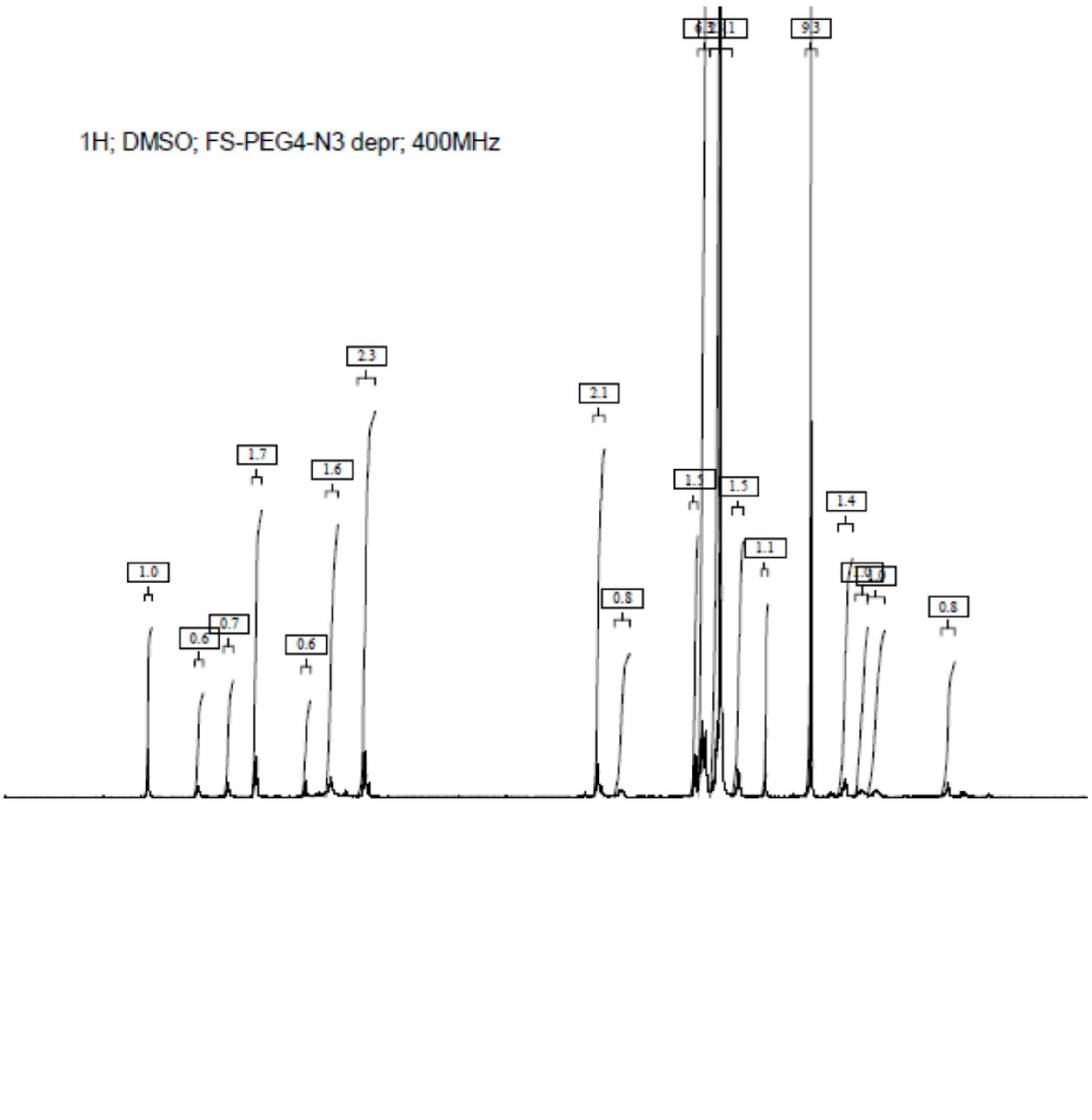

Figure 11. ^{1}H NMR; 400MHz, $CDCl_3$

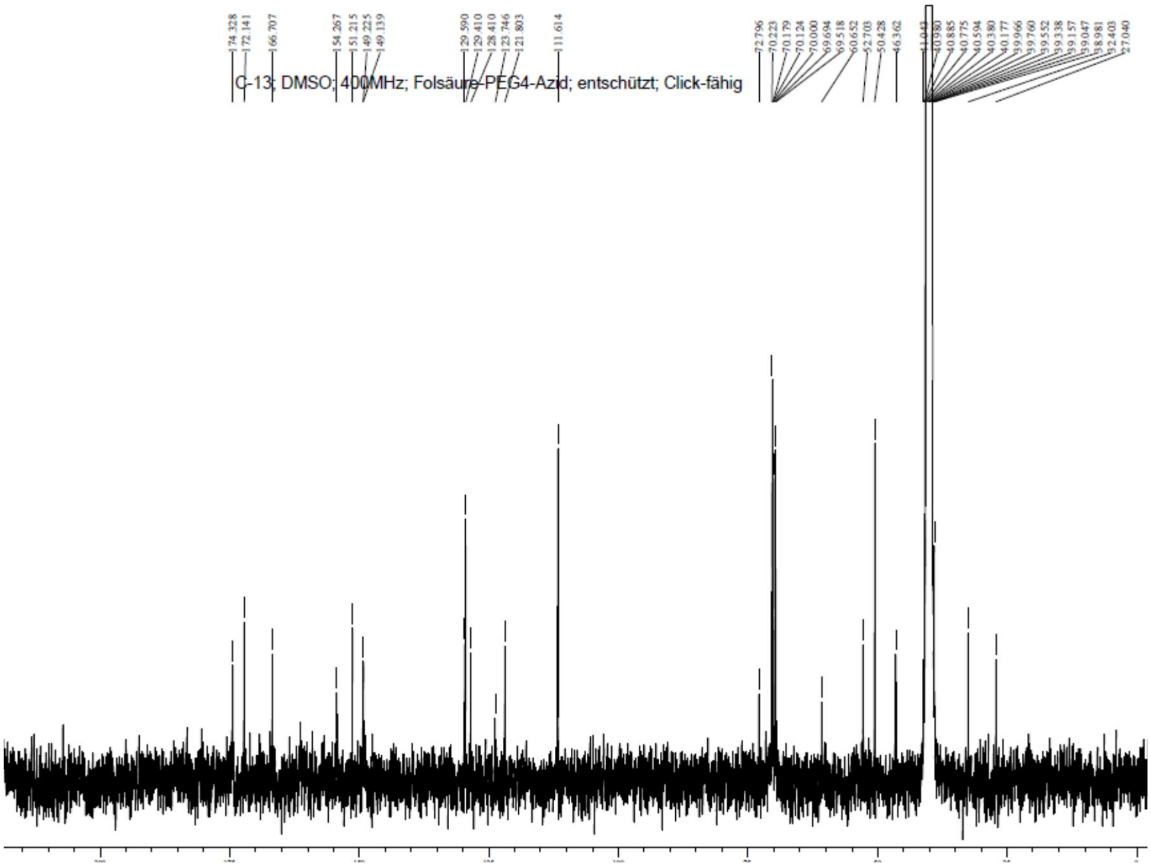

Figure 12. ^{13}C NMR; 400MHz, $CDCl_3$

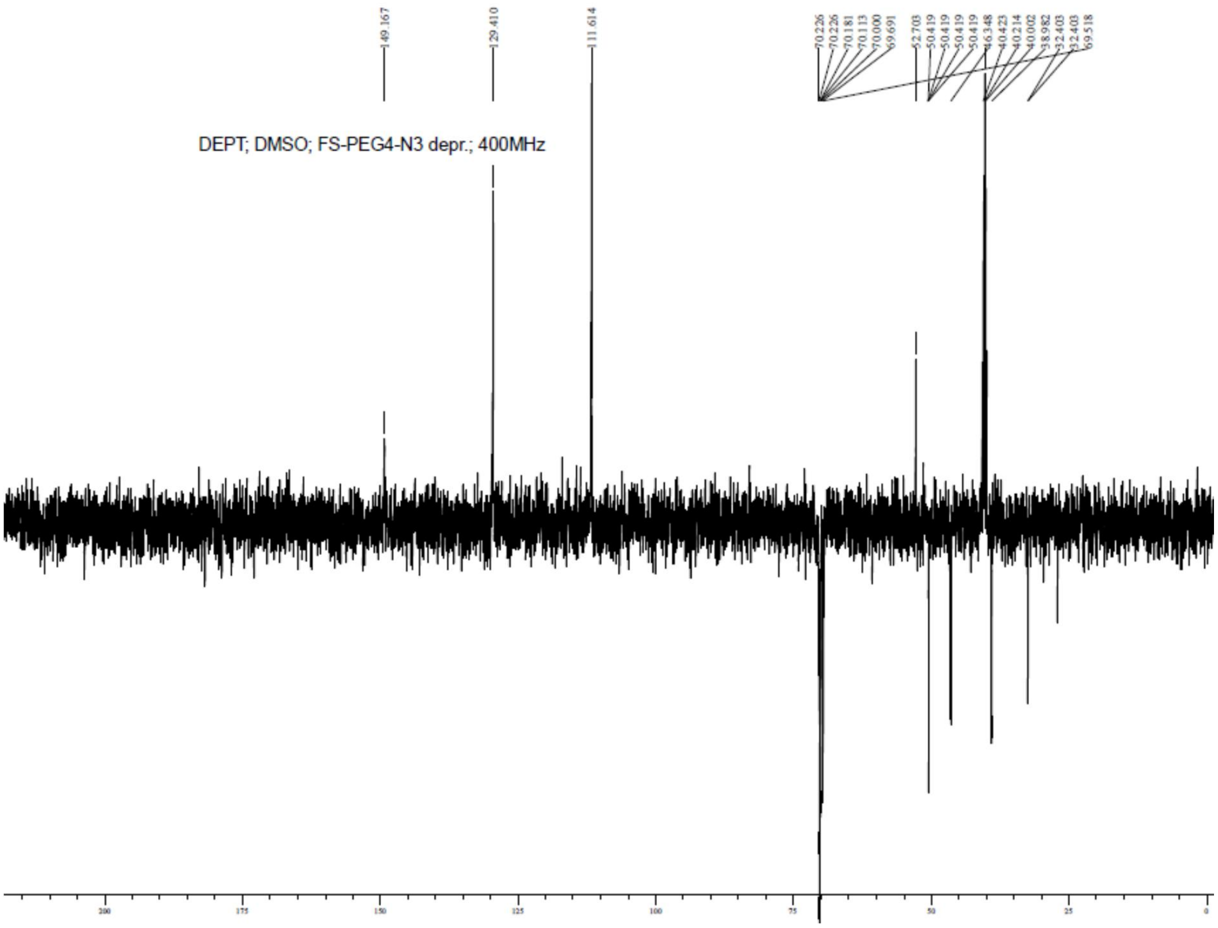

Figure 13. DEPT-135 NMR; 400MHz, $CDCl_3$

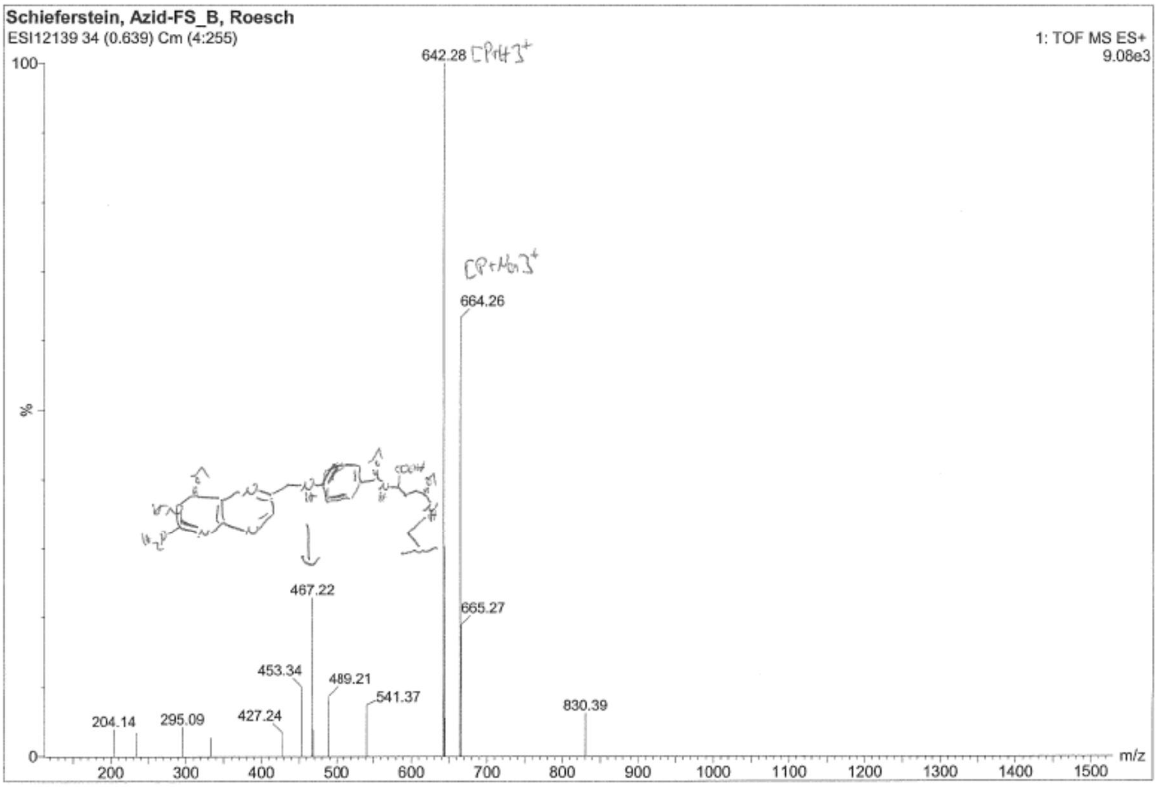

Figure 14. ESI-MS$^+$; NH_4HCO_3-Buffer (0.05 M)

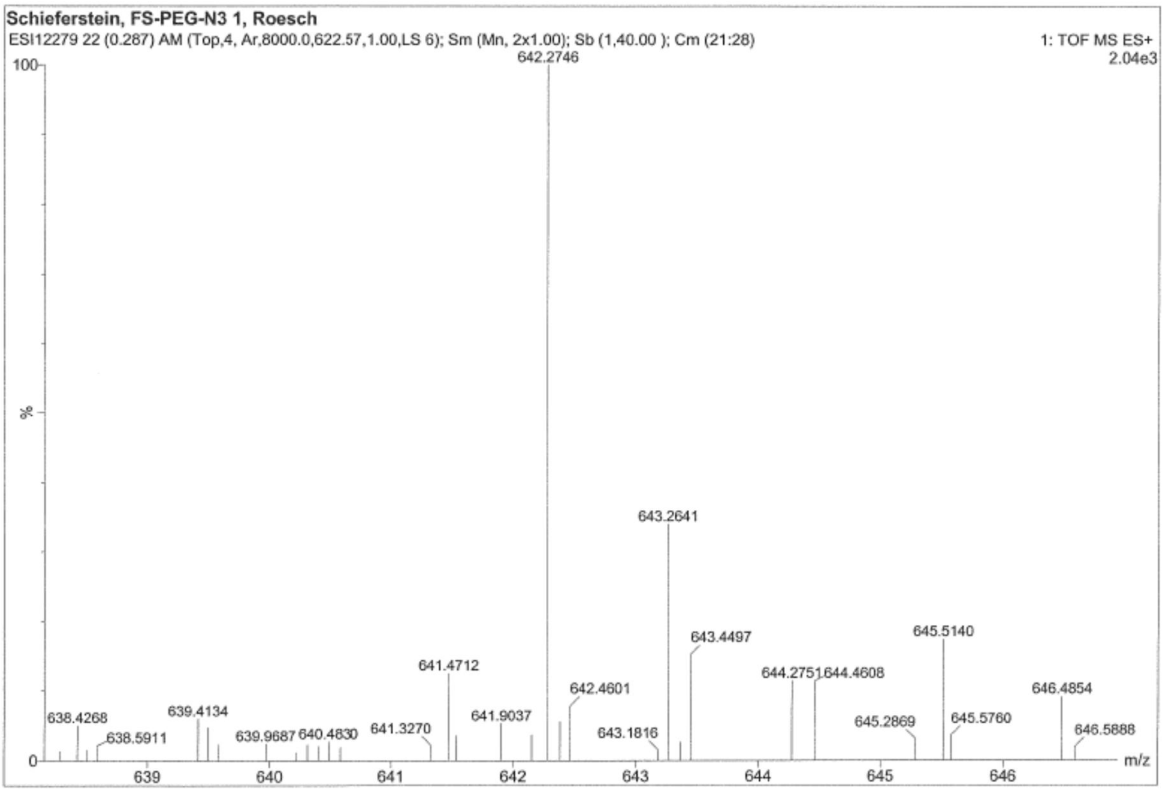

Figure 15. ESI-MS$^+$ (high resolution); NH_4HCO_3-buffer (0.05 M)

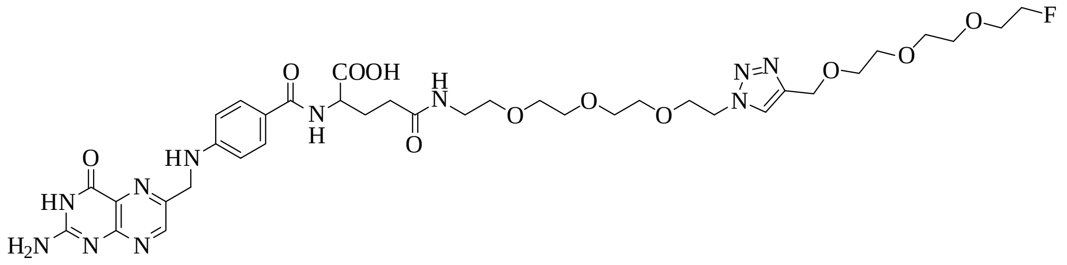

^{1}H NMR (400 MHz, DMSO-d_6) δ 11.58 (br, 1H), 8.63 (s, 1H), 8.18 (d, 1H), 7.93-7.88 (d, 2H), 7.63 (d, 2H), 6.98-6.94 (m, 2H), 6.62 (d, 2H), 4.55 (t, 1H), 4.50 (s, 2H), 4.43 (t, 1H), 4.28-4.22 (m, 1H), 3.87 (t, 2H), 3.65 (t, 2H), 3.58 (t, 2H), 3.55-3.41 (m, 22 H), 3.35 (t, 2H), 3.18-3.12 (m, 2H), 2.20-2.16 (m, 2H), 2.07-1.98 (m, 1H), 1.93-1.84 (m, 1H) ppm. ^{13}C NMR (100 MHz, DMSO-d_6) δ 174.44, 172.13, 166.69, 161.26, 151.22, 149.17, 148.89, 144.88, 129.42, 124.56 121.69, 111.53, 84.42, 82.56, 70.25, 70.21, 70.13, 70.1, 70.03, 69.50, 69.40, 69.14, 64.05, 52.67, 49.74, 46.35, 32.47, 26.73 ppm. LR-MS: $[M+K]^+$ = 870.36 (calc. for $C_{36}H_{50}FKN_{11}O_{11}$: 870.33), $[M+2K]^+$ = 908.32 (calc. for $C_{36}H_{49}FK_2N_{11}O_{11}$: 908.29), $[M+2K]^{2+}$ = 454.65 (calc. for $C_{36}H_{49}FK_2N_{11}O_{11}$: 454.15).

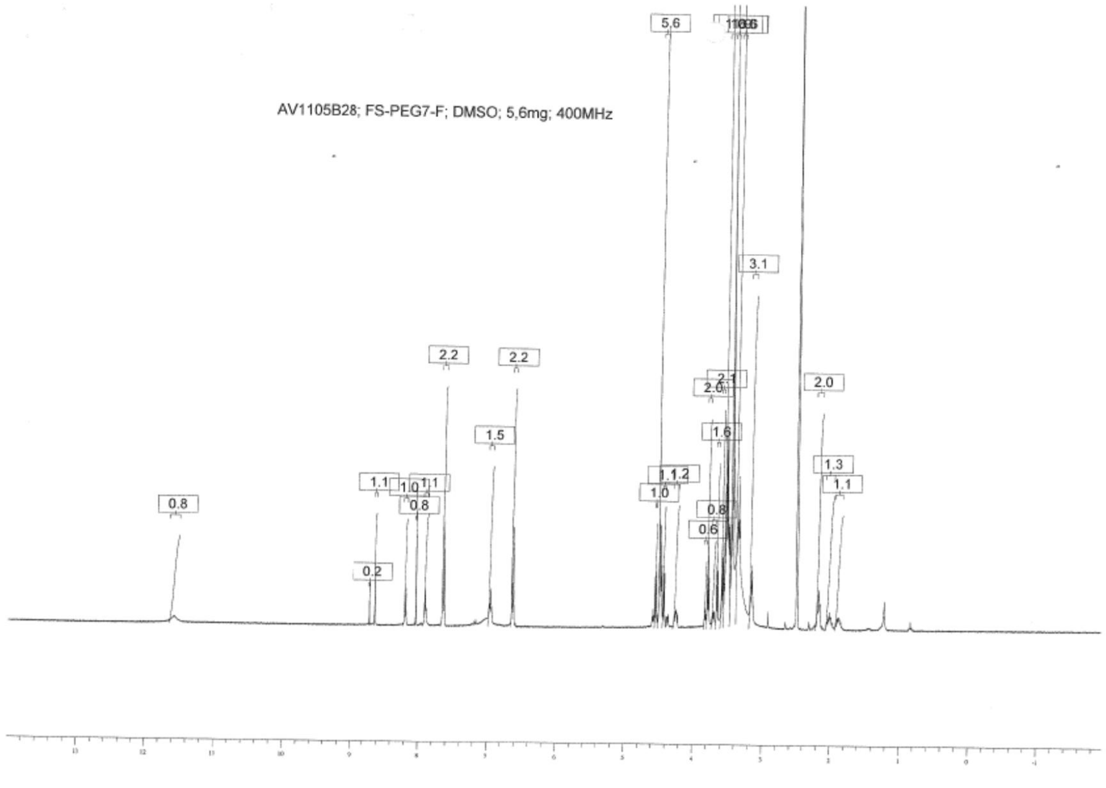

Figure 16. ^{1}H NMR; 400MHz, DMSO-d_6

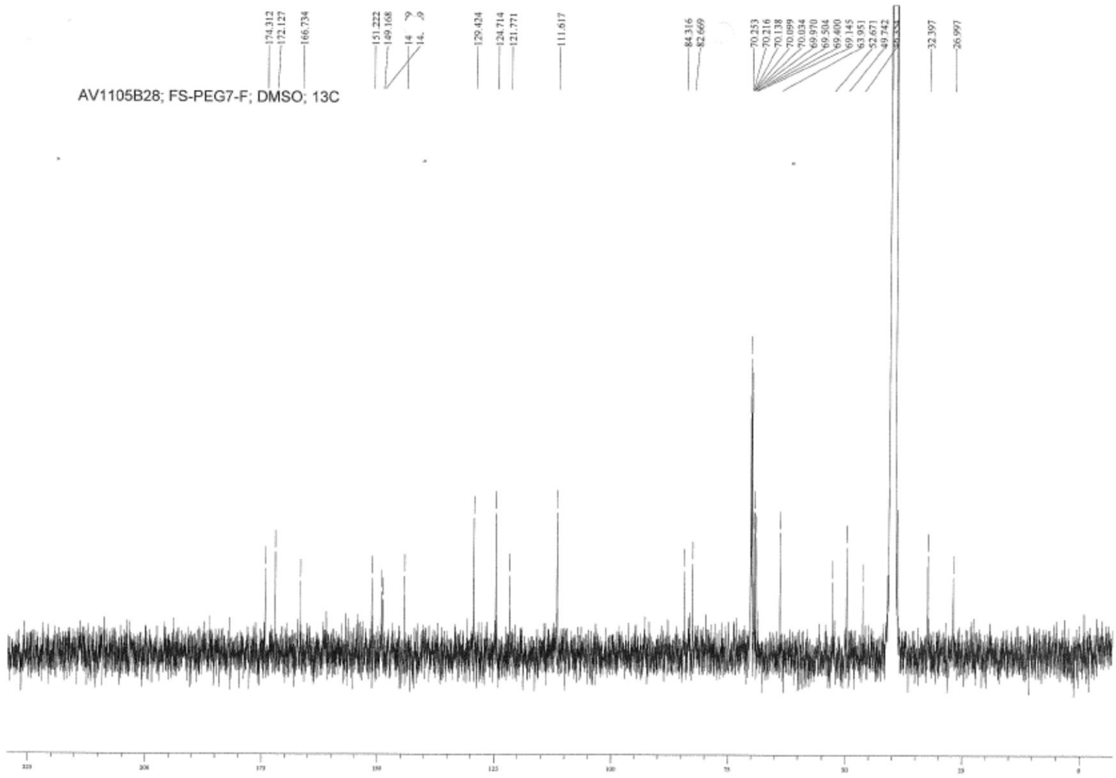

Figure 17 ^{13}C NMR; 400MHz, DMSO-d_6

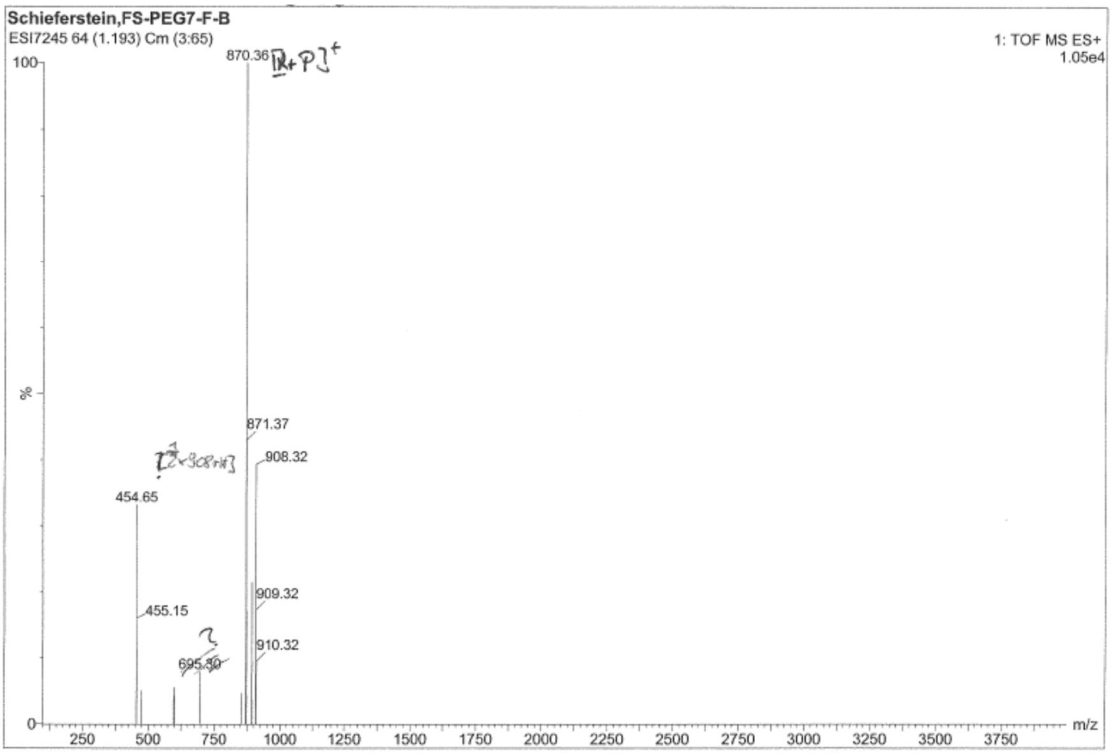

Figure 18. ESI-MS$^+$; Na/K-phosphate-buffer (0.05 M)

Radiochemistry

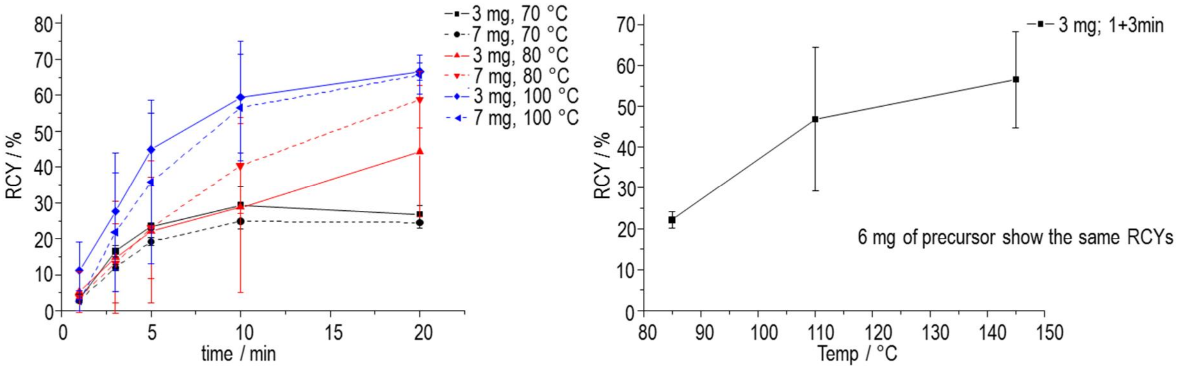

Figure 19. Optimization of the radiolabeling of the prosthetic group. Reaction was performed using acetonitrile and TBA-OH as bas. Conventional heating on the left side and the µwave supported synthesis on the right side.

Prosthetic group synthesis

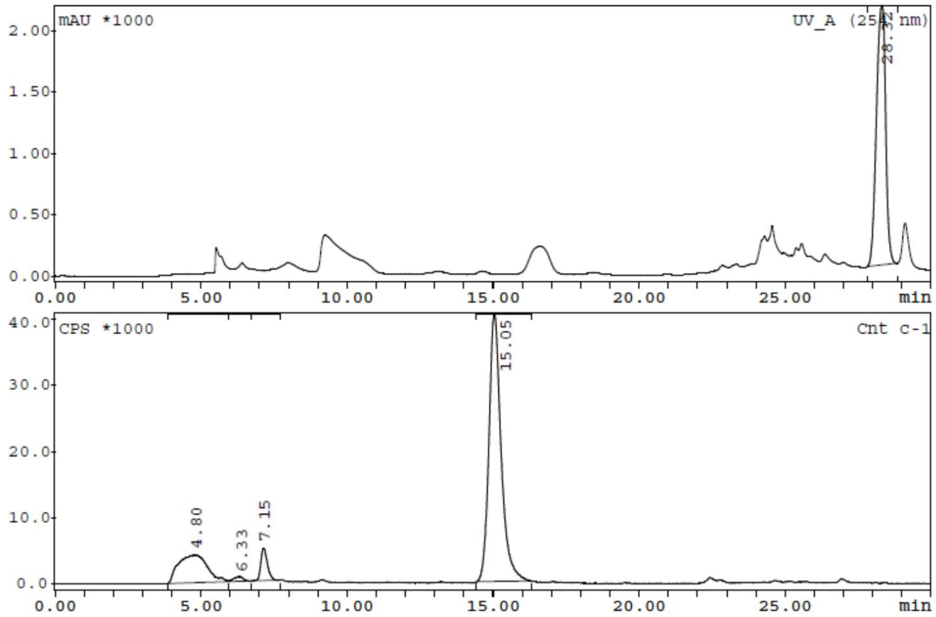

Integration Cnt c-1

Substance	R/T min	Type	Area Counts	%Area %
Reg #1	4.80	BD	287528	18.61
Reg #2	6.33	DD	15000	0.97
Reg #3	7.15	DB	74406	4.82
Reg #4	15.05	BB	1167944	75.60
Sum in ROI			1544877	
Area			1931345	
Ext. BKG			0.00 CPS	

Integration UV_A (254 nm)

Substance	R/T min	Type	Area mAU*min	%Area %
Reg #1	28.32	BB	46365.18	100.00
Sum in ROI			46365.18	

[^{18}F]OEG-folate synthesis

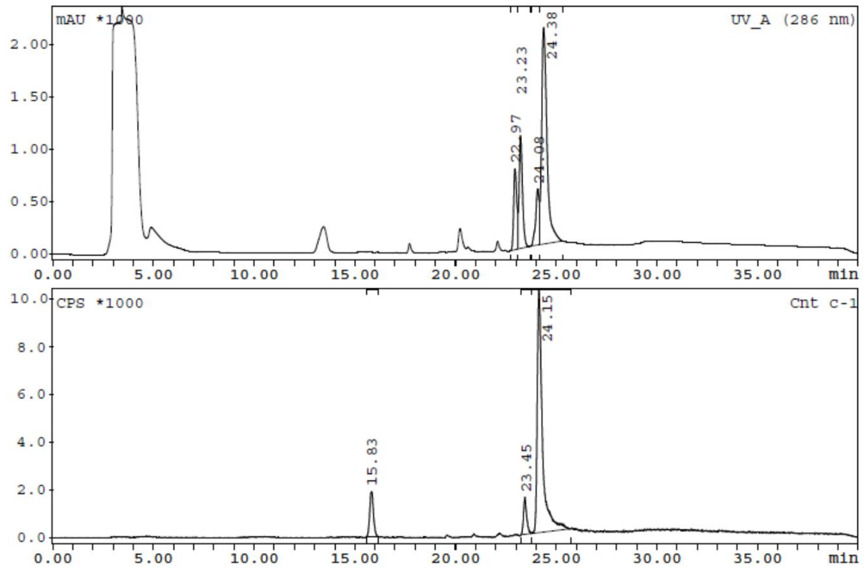

Integration Cnt c-1

Substance	R/T min	Type	Area Counts	%Area %
Reg #1	15.83	BB	24249.9	10.82
Reg #2	23.45	BD	16478.8	7.35
Reg #3	24.15	DB	183438.2	81.83
Sum in ROI			224166.9	
Area			530601.0	
Ext. BKG			0.00 CPS	

Integration UV_A (286 nm)

Substance	R/T min	Type	Area mAU*min	%Area %
Reg #1	22.97	BD	8101.82	12.30
Reg #2	23.23	DB	12854.45	19.51
Reg #3	24.08	BD	6307.47	9.57

Quality Control

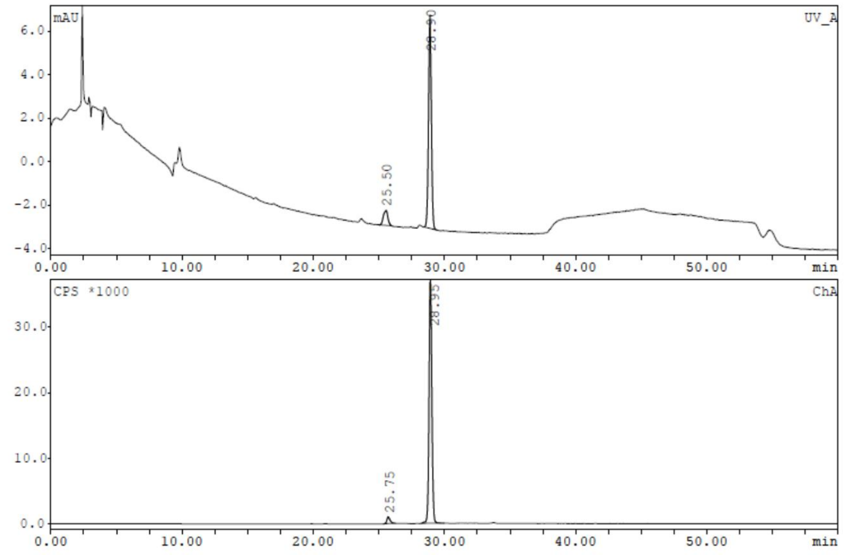

Integration ChA

Substance	R/T min	Type	Area Counts	%Area %
Reg #1	25.75	BB(	16888.9	2.95
Reg #2	28.95	BB(	555790.3	97.05
Sum in ROI			572679.1	
Area			692413.2	
Ext. BKG			0.00 CPS	

Integration UV_A

Substance	R/T min	Type	Area mAU*min	%Area %
Reg #1	25.50	BB(	16.4580	9.97
Reg #2	28.90	BB(	148.5847	90.03
Sum in ROI			165.0427	

Coinjection

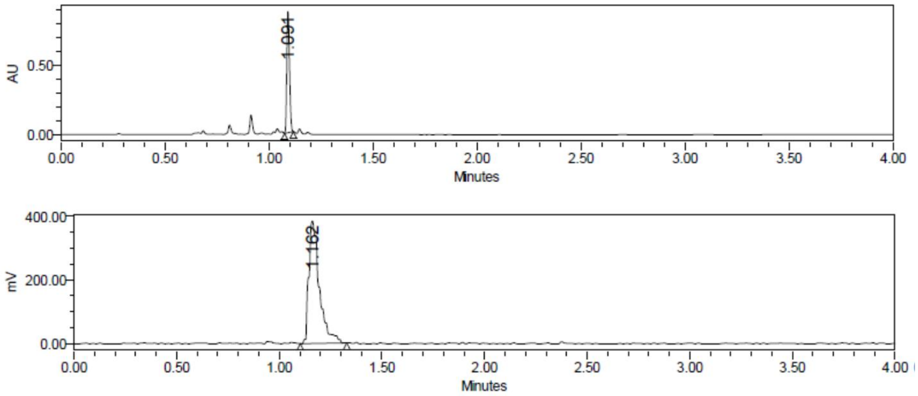

Channel Description: Channel 1, PDA Ch1 254nm@1.2nm

	RT	Area	% Area	Height
1	1.091	784850	100.00	874810

Channel Description: Channel 1

	RT	Area	% Area	Height
1	1.162	1376165	100.00	384444

Biology

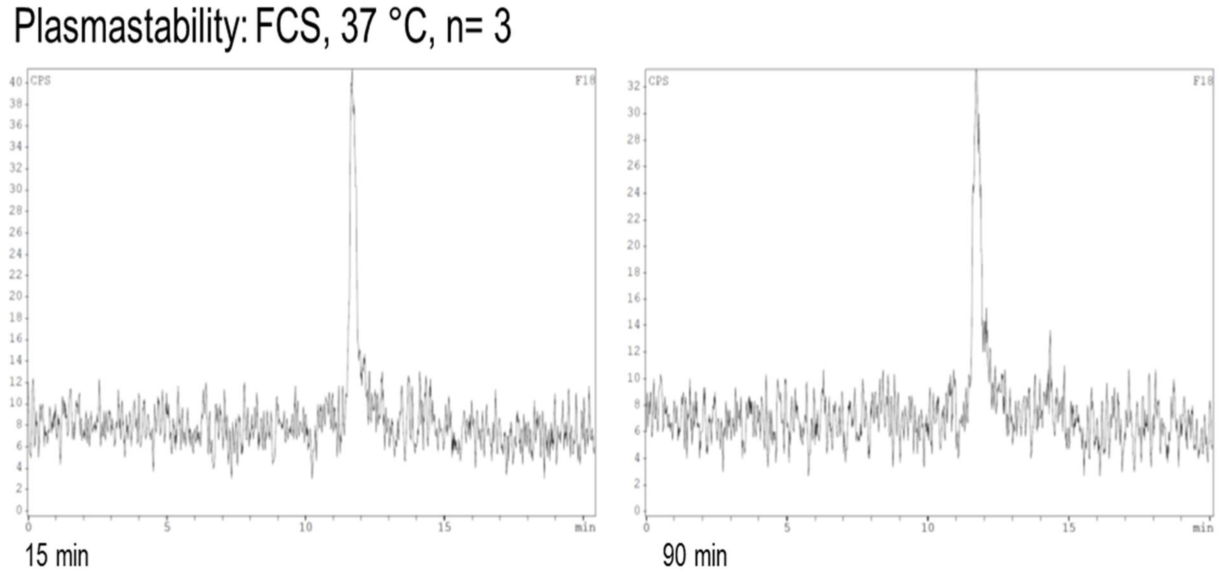

Figure 20. Plasmastability tests using fetal calve serum. Tracer was incubated over 90 min at 37 °C. Aliquots were taken after various time points and injected into an analytical HPLC-system after plasmaproteins have been precipitated.

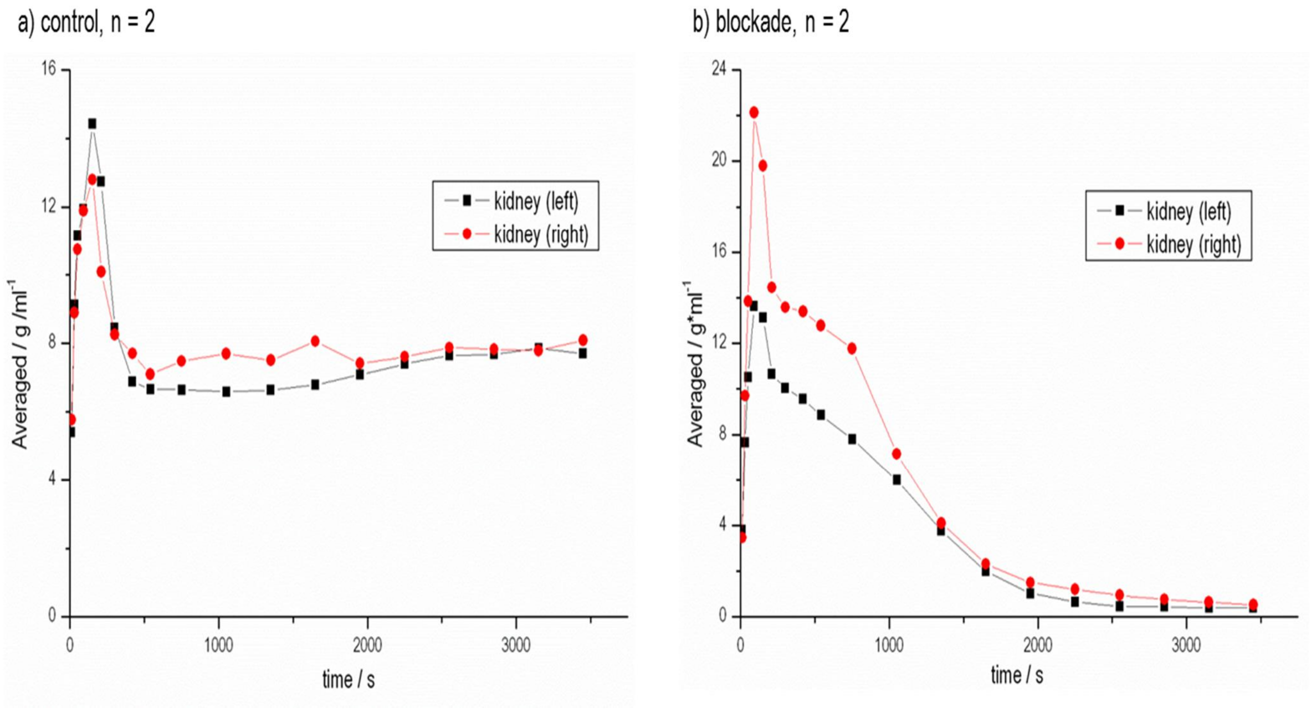

Figure 21. Preliminary µPET studies. left) male healthy CD rats (230 – 310 g), ID 20 – 30 MBq, scanning 60 min dynamic; right) blockade 4 mg/kg 10 min before tracer administration.
